# Extracellular microRNAs modulate human microglial function through TLR8

**DOI:** 10.3389/fimmu.2025.1645062

**Published:** 2025-11-14

**Authors:** Hannah Weidling, Edyta Motta, Leonard D. Kuhrt, Christina Krüger, Caio Andreeta Figueiredo, Thomas Wallach, Silke Frahm, Sebastian Diecke, Susanne A. Wolf, Helmut Kettenmann, Seija Lehnardt

**Affiliations:** 1 Institute of Cell Biology and Neurobiology, Charité – Universitätsmedizin Berlin, corporate member of Freie Universität Berlin, Humboldt-Universität zu Berlin, and Berlin Institute of Health, Berlin, Germany; 2 Max-Delbrueck-Center for Molecular Medicine in the Helmholtz Association, Berlin, Germany; 3 Technology Platform for Pluripotent Stem Cells, Max Delbrück Center for Molecular Medicine in the Helmholtz Association, Berlin, Germany; 4 Centre for Trauma- and Reconstructive Surgery, Charité – Universitätsmedizin Berlin, Corporate member of Freie Universität Berlin, Humboldt-Universität zu Berlin, and Berlin Institute of Health, Berlin, Germany; 5 Institute of Inflammation and Neurodegeneration, Health Campus Immunology, Infectiology and Inflammation (GC-I3), Otto-von-Guericke University, Magdeburg, Germany; 6 Department of Ophthalmology, Charité – Universitätsmedizin Berlin, corporate member of Freie Universität Berlin, Humboldt-Universität zu Berlin, and Berlin Institute of Health, Berlin, Germany; 7 DZHK (German Centre for Cardiovascular Research), partner site Berlin, Berlin, Germany; 8 Shenzhen University of Advanced Technology, Shenzhen Key Laboratory of Immunomodulation for Neurological Diseases, Shenzhen, China; 9 Neuroscience Research Center, Charité – Universitätsmedizin Berlin, corporate member of Freie Universität Berlin, Humboldt-Universität zu Berlin, and Berlin Institute of Health, Berlin, Germany; 10 Department of Neurology, Charité – Universitätsmedizin Berlin, corporate member of Freie Universität Berlin, Humboldt-Universität zu Berlin, and Berlin Institute of Health, Berlin, Germany

**Keywords:** extracellular microRNA, human microglia, microglial function, toll-like receptor 8, stem cells, iPSC, Alzheimer's disease, glioma

## Abstract

**Objective:**

MicroRNAs (miRNAs) are abundantly expressed in the brain and are specifically dysregulated in central nervous system (CNS) diseases. They act as post-transcriptional gene regulators but can also serve as ligands for Toll-like receptors (TLRs). This study aims to investigate CNS disease-associated miRNAs as signaling molecules for human microglia.

**Methods:**

Using a machine learning algorithm and the disease-linked database PhenoMiR, we identified Alzheimer’s disease (AD)- and glioma-associated miRNAs as ligands for TLR7 and TLR8. Expression of human TLR7 and TLR8 in iPSC-derived human microglia-like cells (iMGLs) was validated by RT-qPCR. Using ELISA, scratch assay, and FACS, we investigated the miRNAs’ potential to modulate iMGL function, including cytokine release, motility, and phagocytosis, respectively. The selective human TLR8 antagonist CU-CPT9a was used to determine the role of this receptor in miRNA-induced modulation of human microglial activity. Co-cultures of iMGLs and iPSC-derived human cortical neurons (iNeurons) were analyzed by Neurotrack imaging to assess the effects of miRNAs on human neurites.

**Results:**

We identified AD- and glioma-associated miR-9-5p, miR-132-5p, miR-340-3p, miR-30e-3p, miR-501-3p, and *let-7b* as ligands for human TLR7 and TLR8. Exposure of iMGLs to select miRNAs, including miR-9-5p, miR-132-5p, and miR-340-3p, led to interleukin-6 (IL-6) and tumor necrosis factor (TNF) mRNA expression and protein release in a sequence-dependent fashion. Also, these miRNAs acting as signaling molecules, modulated iMGL motility and phagocytosis activity. The miRNA-induced effects on iMGLs were abolished by CU-CPT9a. Extracellular delivery of miR-132-5p and miR-9-5p to co-cultures of iNeurons and iMGLs resulted in reduced neurite length.

**Discussion:**

Our data establish that distinct CNS disease-associated miRNAs serve as signaling molecules for human microglia via TLR8, thereby controlling the diverse microglial functions and modulating the neuroinflammatory response.

## Introduction

Microglia, the central nervous system’s (CNS) major immune cells, are essential for preserving brain homeostasis, including regulating neurogenesis and synaptic pruning (1–3). They constantly survey their environment, are the professional phagocytes of the brain and can influence the other brain cells by the release of soluble factors (4). Influenced by various environmental cues, microglia can adopt a range of functional states, expanding their role beyond the traditional pro-inflammatory and anti-inflammatory classifications (4, 5).

In brain pathology (6), microglia are critical modulators, particularly in driving inflammatory processes, which are central in various human neurological disorders, such as Alzheimer’s disease (AD) (7), multiple sclerosis (8, 9), and glioma (10). In AD, microglia accumulate around amyloid-beta (Aβ) plaques in an effort to degrade them. As a result, they acquire a proinflammatory phenotype which results in increased secretion of proinflammatory cytokines like interleukin-1-beta (IL-1ß), IL-6, IL-12 and TNF, ultimately leading to neuronal damage and further Aβ accumulation (11–13). Recent studies have also identified risk genes for AD, such as *TREM2*, that are microglia-specific, further underscoring the crucial role of these cells (12). In glioma, tumor-associated microglia are converted to a phenotype with pro-tumorigenic features by the tumor microenvironment through chemokines such as CXCL1, and targeting these microglia can reduce tumor growth (10, 14).

Some microglial inflammatory responses are mediated by Toll-like receptors (TLRs). These membrane receptors belong to a family of pattern recognition receptors, which recognize both pathogen-associated molecules, such as bacterial and viral components, and damage-associated molecules derived from dying cells and tumor tissue (15). Upon activation, TLRs signal through a complex signaling pathway resulting in activation of the interleukin-1 receptor-associated kinases (IRAK), nuclear factor kappa-light-chain-enhancer of activated B cells (NF-κB) and interferon regulatory factor 7 (IRF7) (16, 17). This leads to the production of inflammatory molecules (18), including interferons, proinflammatory interleukins, like IL-1β, IL-6, TNF, and chemokines (19). In microglia, TLR activation modulates motility, phagocytosis of Aβ (20, 21) and drives neuroinflammation by increased cytokine release and NLRP3 inflammasome assembly, further perpetuating neurodegeneration in AD (22).

TLR7 and TLR8 have gained significant attention due to their role as potential therapeutic targets in autoimmune diseases, vaccination adjuvants and targeted immunotherapy in cancer (23–26). Traditionally studied in the context of antiviral immunity (27), these single-stranded RNA (ssRNA) sensors have additionally been shown to mediate neuroinflammatory states in mice, particularly controlling microglial chemotaxis, phagocytosis, and contributing to neuronal injury (20, 28, 29). Additionally, TLRs, particularly TLR7 and TLR8, present notable interspecies differences. While TLR8 in mice was previously considered nonfunctional, murine TLR13 was discussed as the functional homologue to human TLR8. However, recent evidence indicates that murine TLR8 has distinct functional properties (18, 30). This underscores the importance of studying TLR7 and TLR8 in human experimental models within the context of neurological disease.

MicroRNAs (miRNAs) are short, single-stranded RNA molecules that are approximately 22 nucleotides in length. Canonically, they regulate gene expression by binding to complementary messenger RNA (mRNA) (31). miRNAs are widely expressed in the brain, where they regulate homeostasis (32, 33). Specific expression patterns have been associated with neurological disorders (34, 35). In neurodegenerative diseases, miRNA dysregulation has been associated with disease progression by targeting disease-related genes or modulating microglial function (32, 33, 36–38). For example, miR-9 regulates apoptosis, tau hyperphosphorylation, and Aβ accumulation (39), while miR-132 dysregulation correlates with cognitive impairment (40, 41). Additionally, cancer cells have been reported to secrete miRNA via exosomes (42, 43), which suggests a mechanism for intercellular communication that can alter the functional states of microglia (32), further promoting tumor progression by targeting intracellular pathways, such as NF-κB (37, 43, 44). Certain miRNAs have recently been demonstrated to not only regulate gene expression at the post-transcriptional level but also to act as ligands for TLRs, such as TLR7 and TLR8, thereby serving as signaling molecules in the murine CNS (28, 33). These miRNAs acting as TLR ligands include miR-9-5p, miR-132-5p, miR-340-3p, miR-30e-3p, miR-501-3p, and let-7b (28, 45–48). It is likely that more miRNAs serving as ligands for ssRNA-sensing receptors will be identified. Accordingly, miRNAs are detectable in the extracellular space, either derived from dying cells or being actively released from cells via vesicles (28). Importantly, miRNAs bind to TLRs based on sequence specificity. Particularly, GU-rich motifs present in the respective miRNAs are responsible for receptor recognition (27, 28).

Given the growing interest in TLRs and miRNAs as potential therapeutic targets and tools, especially in neurological disease (49–56), alongside the expanding research on miRNA-induced TLR activation observed in murine models, and the noteworthy interspecies differences between murine and human TLRs (16, 18, 27, 30), we addressed the question of whether miRNAs can act as ligands for TLR7 and TLR8 in human microglia. To this end, we generated human microglia-like cells (iMGLs) from induced pluripotent stem cells (iPSCs) (57) and tested their response to the extracellularly applied human miRNAs miR-9-5p, miR-132-5p, miR-340-3p, miR-30e-3p, miR-501-3p, and let-7b. These miRNAs have been previously described to be dysregulated in AD (34, 58–64) and glioma (65–73) and our group has previously confirmed their roles as TLR7/8 ligands in murine microglia (46–48). Our objective was to translate those earlier findings into a human context to enhance the understanding of the extracellular miRNAs’ role in neuroinflammation in the context of human diseases.

## Materials and methods

### Materials

All reagents used in this study are shown in **Supplementary Tables S1, S2**.

### Ethics approval

Conducting research on the iPSC lines was approved by the ethics committee of the Medical Faculty of the University Hospital of the University of Tübingen (approval number 130/2018BO2) and the administrative Panel on Human Subjects in Medical Research, Stanford University (David Spiegel, M.D. 350 [Panel: 3)].

### iPSC lines and culture

Human iPSC lines BIHi268-A-10 (https://hpscreg.eu/cell-line/BIHi268-A-10) were derived from fibroblasts of a healthy female donor and were originally obtained from Helmholtz Zentrum München, Munich, Germany, and further edited and provided by Berlin Institute of Health (BIH, Berlin, Germany). The BIHi005-A-24 (https://hpscreg.eu/cell-line/BIHi005-A-24) cell line is a subclone from BIHi005-A, which was derived from fibroblasts of a healthy man and provided by the BIH. The original BJFF.6 hiPSCs (RRID: CVCL_VU02, www.cellosaurus.org/CVCL_VU02) were generated from commercially available human foreskin fibroblasts (Stemgent, Reprocell, MD, USA.).

The cell lines BIHi268-A-10 and BIHi005-A-24 were maintained in custom-made Essential 8 Medium consisting of DMEM/F12 HEPES (Thermo Fisher, #11330032, Waltham, MA; USA) supplemented with L-ascorbic acid 2-phosphate (Sigma, #A8960, St. Louis, MO, USA), insulin (CS Bio, #C9212-1G, Silicon Valley, CA, USA or Sigma, #91077C-1G, St. Louis, USA), human transferrin (Sigma,#T3705-1G), sodium selenite (Sigma, #S5261-10G), bFGF (PeproTech, #100–18B, Cranbury, NJ, USA), TGFβ1 (PeproTech, #100–21C, Cranbury, USA) and sodium bicarbonate 7.5% solution (Fisher Scientific, #25080–094, Waltham, USA) according to Chen et al. (2011) (74). Two wells of a 6-well cell culture dish were coated with Geltrex (0.12-0.18 mg/ml, Thermo Fisher, #A1569601, Waltham, USA) at 37 °C, 5% CO_2_, 5% O_2_ and passaged every 3–4 days in ratios of 1:6 and 1:12 if they reached approximately 70% confluency using StemPro Accutase (Life Technologies #A11105-01, Waltham, USA), and supplemented with 0.5 μM thiazovivin (STEMCELL Technologies, Vancouver, BC, Canada) for the first 24 h after passaging.

IPSC line BJFF.6 was incubated in StemMACS iPS-Brew XF medium (Miltenyi Biotec #130-107-086, Bergisch Gladbach, Germany) and passaged as described above. hiPSCs were frozen in Bambanker (GC Lymphotec, #302–14681 Tokyo, Japan) for long-term liquid nitrogen storage.

### Quality control

Before being used for microglia or neuron differentiation, all hiPSC lines were thoroughly tested for their quality by the technology platform pluripotent stem cells at Max Delbrueck Center, Berlin. This includes sterility tests via antibiotic-free culture and mycoplasma test (Venor^®^GeM qOneStep Minerva Biolabs, #11–91025 Berlin, Germany), as well as assessment of viability and morphology by phase contrast microscopy. Furthermore, no major structural aberration or chromosomal copy number imbalances were detected by virtual SNP-karyotyping using Illumina OMNI-EXPRESS-8v1.6 Chip (Illumina, San Diego, CA, USA). Cell line identity was confirmed by short tandem repeat (STR) analysis using GenePrint^®^ 10 System (Promega, #B9510, Fitchburg, MA, USA).

### Differentiation of iPSCs into iMGLs

Differentiation of iPSCs to hematopoietic progenitor cells (HPCs) was performed following the protocol by McQuade et al. (57). In brief, human induced pluripotent stem cells (iPSC BIHi-268-A-10 and BJFF.6) were cultured for a maximum of three weeks as described above until they reached 80% confluency. Afterwards, cells were passaged with ReLeSR (STEMCELL Technologies #100-0484, Vancouver, Canada) into TeSR-E8 medium with 0.5 μM Thiazovivin (STEMCELL Technologies #72252, Vancouver, Canada) onto Geltrex-coated 6-well plates (Corning, #353046, Corning, NY, USA). After 24 h, when approximately 60–100 cell colonies have been achieved, TeSR-E8 medium was replaced with 2 ml/well STEMdiff™Hematopoietic Basal Medium plus Supplement A (1:200 dilution; STEMCELL Technologies #05310, Vancouver, Canada). After 48 h, a half media change was performed. On day 3, complete media was exchanged by 2 ml/well of STEMdiff™ Hematopoietic Basal Medium plus supplement B (1:200 dilution; STEMCELL Technologies #05310, Vancouver, Canada). Without media removal, supplementation with 1 ml/well of medium B was performed on days 5, 7, 9 and 10. On day 12, non-adherent cells were collected and centrifuged at 300 x g for 5 min. Fluorescence-activated cell sorting (FACS) analysis has previously shown that these non-adherent cells represent highly pure populations (> 93%) of CD43^+^ hematopoietic progenitor cells (HPCs) (57).

HPCs were plated at a density of 130,000 cells per well onto Geltrex-coated 6-well plates (Corning, #353046, Corning, USA) in DMEM/F12 without phenol red (ThermoFisher #11039021, Waltham, USA) supplemented with 2X insulin-transferrin-selenite (ThermoFisher #41400045, Waltham, USA), 2X B27 (ThermoFisher #17504044, Waltham, MA, USA), 0.5X N2 (ThermoFisher #17502048, Waltham, USA), 1X Glutamax (ThermoFisher #35050038, Waltham, USA), 1X non-essential amino acids (ThermoFisher #11140035, Waltham, USA), 400 μM monothioglycerol (Merck #M1753, Darmstadt, Germany), and 5 μg/ml insulin (PromoCell, #C-52310, Heidelberg, Germany). Immediately before use, the medium was supplemented with 100 ng/ml IL-34 (Peprotech #200-34, Cranbury, USA), 50 ng/ml TGFβ1 (Peprotech #100-21C, Cranbury, USA), and 25 ng/ml M-CSF (Peprotech #300-25, Cranbury, USA) taken from single-use frozen aliquots. On days 2, 4, 6, 8, and 10, 1 ml fresh media plus freshly thawed cytokine cocktail was added. On day 12, non-adherent cells were collected and centrifuged for 5 min at 300xG. The cell pellet was resuspended in 1 ml fresh differentiation medium per well and placed back in the same well. Media supplementation was continued (1 ml) on days 14, 16, 18, 20, 22, and 24. On day 25, non-adherent cells were centrifuged as on day 12. Cells were resuspended in 1 ml media/well plus 100 ng/ml IL-34, 50 ng/ml TGFβ1, 25 ng/ml M-CSF, 100 ng/ml CD200 (Bon-Opus, #BP004, Millburn, NJ, USA) and 100 ng/ml CX3CL1 (Peprotech #300-31, Cranbury, USA) and added back to the same well. On day 27, 1 ml microglia maturation media with a five-cytokine cocktail was added per well. On the following days, cells were used for experiments.

### Analysis of cytokine expression and release

Cells were harvested and plated at 50,000 cells/well into 150 µl maturation medium in a 96-well plate. The next day, cells were stimulated. LPS (1 µg/ml, Merck, #L43191, Darmstadt, Germany), loxoribine (1 mM, InvivoGen, #tlrl-lox, San Diego, USA), R848 (10 µg/ml, InvivoGen, #tlrl-r848-1, San Diego, USA), and TL8-506 (100 ng/ml, InvivoGen, #tltl-Tl8506, San Diego, USA) served as positive controls. Unstimulated condition, LyoVec complexed with water as a vehicle control, and mutated oligonucleotide (10 µg/ml) were used as negative controls. miRNAs and mutated oligonucleotides were complexed to LyoVec (InvivoGen, San Diego, USA) to a concentration of 10 µg/ml. After 24 h of exposure, supernatants were collected and stored at -80 °C. TNF and IL-6 concentrations in the supernatants were measured by Enzyme-Linked Immunoabsorbent Assay (ELISA) according to the manufacturer’s instructions (TNF alpha Human Uncoated ELISA Kit, Invitrogen, #88–7346-88, Waltham, USA; IL-6 Human Uncoated ELISA Kit, Invitrogen, #88–7066-88, Waltham, USA). Samples were analyzed using the Varioskan Flash device (Thermo Fisher Scientific, Waltham, MA, USA). Furthermore, cells were lysed using the ReliaPrep RNA kit (Promega, #Z6112, Fitchburg, USA), and lysates were stored at -20 °C. Total RNA was isolated using the ReliaPrep RNA tissue kit (Promega, #Z6112, Fitchburg, USA) according to the manufacturer’s instructions. Quality and yield were determined by a NanoDrop 8000 spectrometer (Thermo Fisher, #ND8000LAPTOP, Waltham, USA). cDNA was synthesized using PrimeScript RT reagent kit (Takara Bio, #RR037B Kusatsu, Japan) from 100 ng total RNA whenever possible. Samples with limited RNA input were also processed. RT-qPCR gene amplification was performed using the 7500 Fast Real-Time PCR System (Applied Biosystems, Waltham, USA) with SYBR Green Master Mix (Life Technologies, # 4472918, Waltham, USA) as previously described (75–77). In brief, the total reaction volume was 20 µl, containing 1 ng of cDNA template. For cytokine expression analysis, primers were used at a final concentration of 2 µM each, based on previous studies (75–77). For receptor expression analysis, the primer concentration was reduced to 1 µM each. Specificity for all reactions was confirmed by melting curve analysis and no-template controls. All samples were run in technical duplicates; for samples with sufficient RNA quantity, technical triplicates were performed to ensure reproducibility. The results were analyzed by the 2-^ΔΔCT^ method, normalized to human TATA-box-binding protein (*TBP*) as a housekeeping gene and were presented as fold change normalized to the unstimulated condition. Primer sequences are listed in the additional **Table 1**.

**Table 1:** Selection of miRNAs as potential signaling molecules for human microglia investigated in this study.

miRNA	Associated with
hsa-miR-9-5p UCUUUGGUUAUCUAGCUGUAUGA	AD (34, 58–60) glioma (67, 69, 70)
hsa-miR-340-3p UCCGUCUCAGUUACUUUAUAGC	AD (63) glioma (65, 66)
hsa-miR-132-5p ACCGUGGCUUUCGAUUGUUACU	AD (60) glioma (67)
hsa-miR-30e-3p CUUUCAGUCGGAUGUUUACAGC	AD (60) glioma (68)
hsa-miR-501-3p AAUGCACCCGGGCAAGGAUUCU	AD (60, 61) glioma (67, 71)
hsa-let-7b UGAGGUAGUAGGUUGUGUGGUU	AD (62, 64) glioma (72, 73)

Alzheimer’s disease and glioma-associated miRNAs, as determined by the PhenomiR database (81), with high potential to activate TLR7 and/or TLR8 based on their sequence and structure, were predicted by the *Braindead* algorithm (46). Established hTLR7/8 recognition motifs within the respective miRNA sequence are underlined (29, 46, 47, 111, 112).

### TLR8 inhibitor assay

iMGLs were harvested as stated above. The next day, cells were preincubated with CU-CPT9a (1 mM, InvivoGen, #inh-cc9a, San Diego, USA), a specific TLR 8 inhibitor (78), for 3 h. Afterwards, cells were stimulated with TL8-506 (100 ng/ml, InvivoGen, #tltl-Tl8506, San Diego, USA) and imiquimod (5µg/ml, InvivoGen, #tlrl-imqs-1, San Diego, USA). MiRNAs were complexed to LyoVec (InvivoGen, San Diego, USA) to a concentration of 10 µg/ml. After 24 h of stimulation, supernatants were collected and stored at -80 °C. IL-6 concentrations in the supernatants were measured by ELISA according to the manufacturer’s instructions (IL-6 Human Uncoated ELISA Kit, Invitrogen, #88–7066-88, Waltham, USA).

### Motility assay

iMGLs were harvested and plated at 100,000 cells/well in 100 µl maturation medium into an image lock plate (Essen Bioscience # 4379, Ann Arbor, MI, USA). The next day, a cell-free wound area was created using Incucyte^®^ 96-well WoundMaker Tool (Essen Bioscience, #4563, Ann Arbor, USA). Cells were stimulated with Pam2CSK4 (100 ng/ml, R&D Systems, 4637/1, Minneapolis, USA), a TLR 2 agonist known to induce iMGL motility (75) and otherwise similarly to the cytokine release assay described above. For experiments investigating the effect of TLR8 inhibition, cells were pre-treated with CU-CPT9a and subsequently stimulated as described above with the indicated concentrations. Pictures were taken after 6, 12, 24, 36, or 48 h after stimulation. The covered area was assessed using an ImageJ Plugin (https://github.com/AlejandraArnedo/Wound-healing-size-tool/wiki) (79). The migration rate was calculated according to the following equation:


$$covered\;area=\frac{A_{t0}-A_{x}}{A_{t0}}*100$$

A_t0_= area at start A_x_= area after 36 h.

### FACS-based phagocytosis assay

iMGLs were harvested and plated into a 96-well plate at a density of 50,000/well. On the next day, cells were stimulated with 10 µg/ml of miRNAs, LPS (1 µg/ml, Merck, #L43191, Darmstadt, Germany), or TL8-506 (100 ng/ml, InvivoGen, #tltl-Tl8506, San Diego, USA) for either 5 or 24 h. After incubation time, pHrodo™ Red *E. coli* BioParticles™ (Thermo Fisher, #P35361, Waltham, USA) were added to the medium (4 µg/ml) for 1 h to the cells prestimulated for 5 h, or for 5 h to the cells prestimulated for 24 h. Afterwards, iMGLs were washed and stained for viability using Zombie NIR fixable dye (Biolegend, #423105, San Diego, CA, USA) and fixed in 4% PFA in DPBS for 20 min. Samples were acquired with an LSRFortessa flow cytometer (BD Life Sciences, Franklin Lakes, NJ, USA) and further analyzed with FlowJo™ (v10, LLC, BD Life Sciences, Franklin Lakes, USA).

### Differentiation of iPSC-derived human cortical neurons

iNeurons were differentiated according to the protocol Zhang et al. (2013) (80) with minor changes. In short, on day -1 iPSC (BIHi005-A-24) were split with TrypLE (Life Technologies, A1217701, Waltham, USA) and plated at a density of 5 x 10^5^ cells into a Geltrex-coated 6-well plate (Corning, #353046, Corning, USA) in E8 medium (Thermo Fisher, A1517001, Waltham, USA) containing ROCK inhibitor (0.5 μM Thiazovivin (STEMCELL technologies #72252 Vancouver, Canada 10µM). On day 0 and 1, a full media change was performed with medium containing DMEM/F12 (Life Technologies, 11330-032, Waltham, USA), N2 supplement (100X) (Thermo Fisher, 17502-048. Waltham, USA), NEAA (Life Technologies, 11140-035, Waltham, USA), hBDNF (10 ng/ml, PeproTech, 450-02-10. Cranbury, USA), hNT-3 (10 ng/ml, R&D Systems, 267-N3-025, Minneapolis, USA), laminin (0.2 μg/ml, Sigma Aldrich, #L2020-1MG, St. Louis, USA) and doxycycline (2 µg/ml, Sigma-Aldrich, D9891, St. Louis, USA). On day 2 and 3, a full media change was performed using medium containing Neurobasal medium (Life Technologies, 21103-049, Waltham, USA), B-27 supplement (Thermo Fisher, 17504-044, Waltham, USA), GlutaMAX (Life Technologies, 35050-038, Waltham, USA), hBDNF (10 ng/ml, PeproTech, 450-02-10, Cranbury, USA), hNT-3 (10 ng/ml, R&D Systems, 267-N3-025, Minneapolis, USA), laminin (0.2 μg/ml, Sigma-Aldrich, L2020, #L2020-1MG, St. Louis, USA), AraC (5 μM added only at day 4, Sigma-Aldrich, C1768, St. Louis, USA), doxycycline (2 μg/ml, Sigma-Aldrich, D9891, St. Louis, MO. USA. On days 4-14, we performed a half media change with NB-B27 medium. On day 8, we performed a TrypLE single cell split to split cells on a Geltrex-coated 96-well plate suitable for imaging (Cellvis, P96-1.5P, Mountain View, CA, USA) at a density of 65,000 cells/well.

### Co-culture of iMGLs and iNeurons

iMGLs and iNeurons were differentiated, as described above. On day 14 or 15 of neuron differentiation, iMGLs were added at a ratio of 1:3 (iMGLs:iNeurons), and cells were left to rest overnight in iMG differentiation medium without cytokines except 25 ng/ml M-CSF (Peprotech #300-25, Cranbury, USA). On the next day, cells were stimulated with miRNAs miR-9-5p and miR-132-5p, and TLR agonists, TLR agonists LPS and TL8-506. miRNAs were complexed to LyoVec (InvivoGen, San Diego, USA) to a concentration of 10 µg/ml, added to the co-culture in an Incucyte system (Sartorius, Incucyte^®^ SX5, Göttingen, Germany) for 5 d. LPS (1 µg/ml, Merck, #L43191, Darmstadt, Germany) and TL8-506 (100 ng/ml, InvivoGen, #tltl-Tl8506, San Diego, USA) served as positive controls, while the unstimulated condition was used as a negative control.

### Quantification of iNeuron neurite length

Images of cell cultures in the Incucyte system, as described above, were taken every 2 h using the built-in 4x microscope. After 5 days, the supernatant was frozen and stored at -80 °C. Images were analyzed using the Incucyte Neurotrack™ Analysis software according to the company’s instructions (Sartorius, Incucyte^®^ SX5, Göttingen, Germany). Relative change of neurite length was calculated using the following equation: $\frac{{\text{l}}_{\text{x }}-l_{0}}{l_{0}}*100$, in which l_x_ stands for neurite length after 2 days, and l_0_ equals neurite length at the start of the experiment.

### Statistical analysis

Data are expressed as mean ± SEM if normally distributed and as median ± interquartile range if not normally distributed. For comparison of data between more than two experimental groups, a combination of one-way ANOVA followed by Dunnett *post-hoc* test, Holm-Sidak’s *post hoc* test or Tukey multiple comparison test was used when a normal distribution was present, and Kruskal-Wallis followed by Dunn’s *post hoc* test for multiple comparisons when no normal distribution was present. Mann-Whitney-U test was used to compare data between two experimental conditions when applicable, followed by Benjamini-Hochberg correction for multiple testing. GraphPad Prism 9 (GraphPad Software, San Diego, CA, USA) was used for statistical analysis. Information about the statistical tests and the number of experiments is given in the figure legends. Significance levels are indicated as n.s., not significant; *p < 0.05; **p< 0.01; ***p< 0.005; ****p < 0.001.

## Results

### Identifying miRNAs as potential signaling molecules for RNA-sensing TLRs in iMGLs

We aimed to investigate miRNAs associated with AD and glioma as potential signaling molecules for human microglia. To this end, we made use of our previously published datasets (46), derived from our recently developed machine learning approach, *Braindead*, which predicts small RNAs as potential ligands for TLR7 and TLR8, based on the RNA’s sequence and structure (29, 46). A subset of miRNAs predicted to be ligands for human (h)TLR7 and/or hTLR8 has been validated using HEK hTLR7 and hTLR8 reporter cell systems (29, 46, 47). In our current study, these miRNAs identified as ligands for hTLR7 and hTLR8 were further analyzed with respect to their association with AD and glioma using the disease-linking PhenomiR database (81). Thereby, a list of miRNA candidates was generated, including miR-9-5p, miR-132-5p, miR-340-3p, miR-30e-3p, and miR-501-3p. *Let-7b*, an established miRNA ligand for murine TLR7 (mTLR7) and potent activator of murine microglia (28, 48), was included as a reference miRNA (**Table 1**). All the candidate miRNAs, including *let-7b*, have been previously described to be dysregulated in AD and glioma (34, 58–73). In addition, all these miRNAs are released from apoptotic neurons and are stable in the extracellular space (28, 29, 47). Thus, we considered these candidate miRNAs as potential signaling molecules in the CNS and determined their capability to activate human microglia in the following experimental setups.

### iMGLs express human TLR7 and TLR8

**Figure 1:**
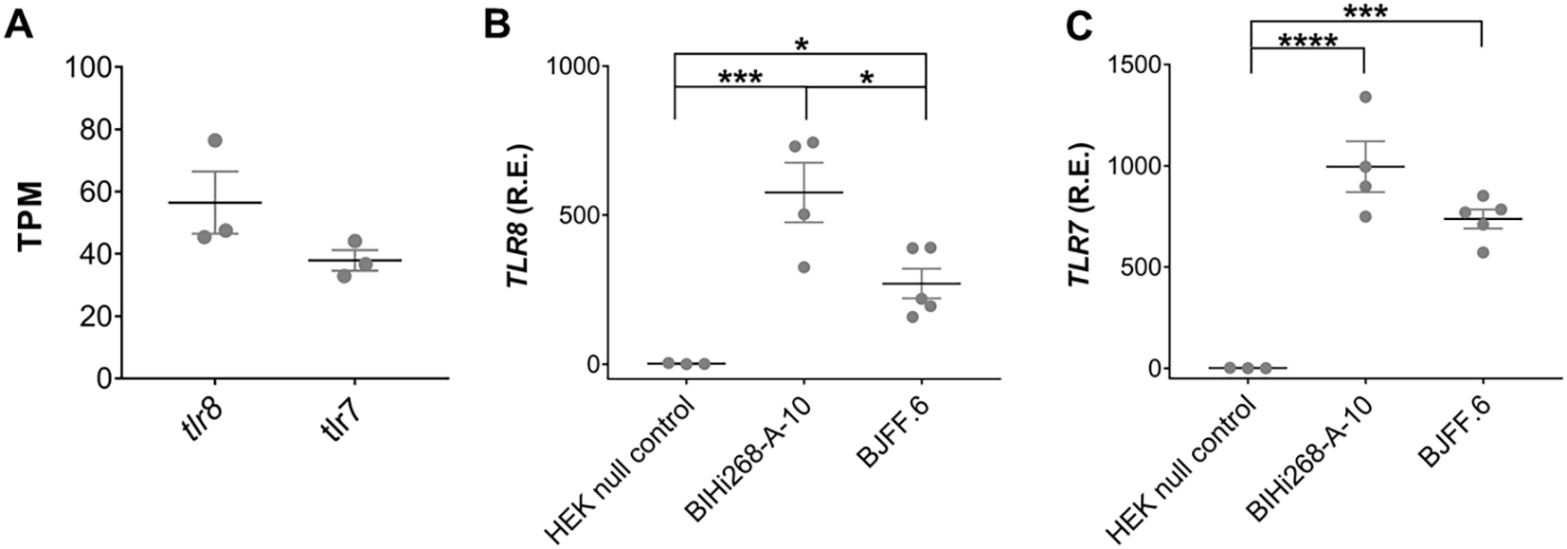
iMGLs express TLR7 and TLR8. **(A)** Re-analysis of *TLR7* and *TLR8* expression in iMGLs from the previously published data set by Abud et al. (82). Data shown as transcripts per kilobase million (TPM). Analysis of relative mRNA expression (R.E.) of *hTLR8***(B)** and *hTLR7***(C)** in induced microglia-like cells (iMGL) generated from the iPSC lines BIHi268-A-10 and BJFF.6 by quantitative reverse transcription-PCR (RT-PCR). Expression is shown based on 2-^ΔΔCT^ values relative to expression of the TATA box binding protein (*TBP*) serving as a house keeping gene. Human embryonic kidney (HEK) 293 *null* cells lacking both *TLR7* and *TLR8* expression, served as negative control. Data are represented as mean (line) with SEM (whiskers) and single data points (dots), and were analyzed using one-way ANOVA followed by Holm-Sidak’s *post hoc* test. *p < 0.05; **p < 0.01; ***p < 0.001; **** = *p*< 0.0001; versus control. *n* = 3-5.

Re-analysis of a publicly accessible RNAseq data set derived from iMGLs, which were differentiated by the same methodology as used in our laboratory (82), revealed that both *hTLR7* and *hTLR8* are expressed in iMGLs (**Figure 1A**). Expression of *hTLR7* and *hTLR8* in iMGLs derived from the two iPSC lines, BIHi268-A-10 and BJFF.6 lines was validated by RT-qPCR (**Figures 1B, C**). HEK293 *null* cells lacking *hTLR7* and *hTLR8* expression (83) were used as a negative control. Of note, the hTLR8 mRNA expression levels in the BIHi268-A-10 line were significantly higher than the expression levels in the BJFF.6 line (**Figures 1B, C**).

### Extracellular miRNAs induce cytokine release in a sequence-dependent fashion

**Figure 2:**
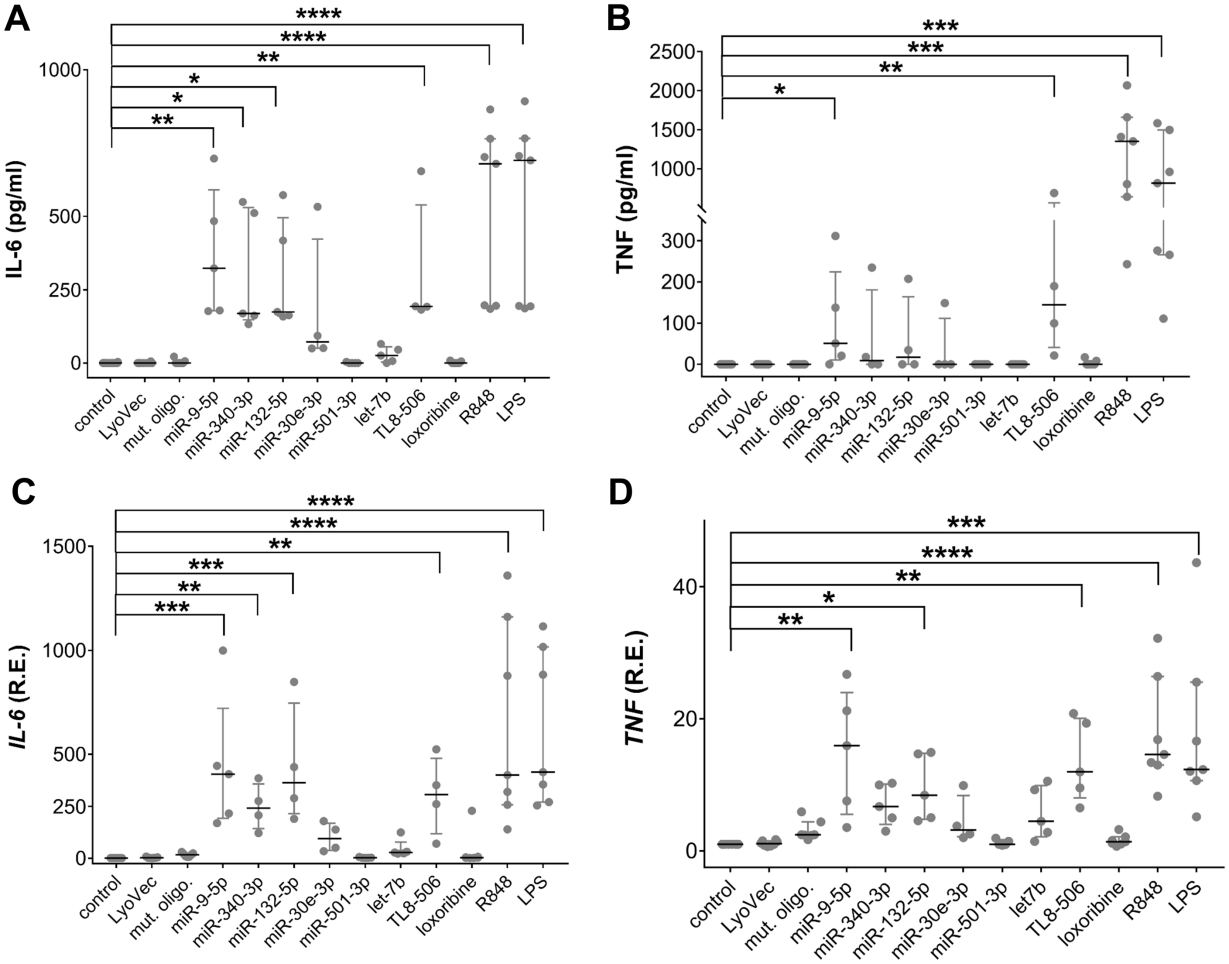
Extracellular miRNAs trigger cytokine release in a sequence-specific fashion. **(A-D)** BIHi268-A-10-derived iMGLs were incubated with 10 µg/ml of miRNAs, as indicated, for 24 h LPS (1 µg/ml), loxoribine (1 mM), R848 (10 µg/ml), and TL8-506 (100 ng/ml) served as control for activation of TLR4, 7, 7/8, and 8, respectively. Unstimulated cells, LyoVec, and mutant oligoribonucleotide (10 µg/ml) served as negative controls. Assessment of IL-6 **(A)** and TNF **(B)** protein concentrations determined by ELISA in supernatant of iMGLs treated as described above. qPCR analysis of *IL-6***(C)** and *TNF***(D)** mRNA expression in iMGLs as described above. *n* = 4–7 individual experiments. Data are shown as a median (line) with an interquartile range (whiskers) and single data points (dots). Kruskal-Wallis test, followed by Dunn’s *post-hoc* multiple comparisons test compared to the unstimulated condition and Mann-Whitney U test, followed by a Benjamini-Hochberg *post hoc* test for multiple testing. *p < 0.05; **p < 0.01; ***p< 0.005; ****p < 0.001, compared to unstimulated condition.

To determine the potential of extracellular human CNS disease-associated miRNAs to modify human microglia, iMGLs differentiated from the BIHi268-A-10 line were exposed for 24 h to the miRNA candidates selected above (see **Table 1**), including miR-9-5p, miR-132-5p, miR-340-3p, miR-30-3p, miR-501-3p, and *let-7b* (**Figure 2**). Lipopolysaccharide (LPS), a TLR4 agonist and an established microglial modulator (84, 85), resiquimod 848 (R848), a dual agonist for TLR7/8 (86), the TLR7 agonist loxoribine (87), and TL8-506, an analog of the hTLR8 agonist Motolimod (88), were used as references for TLRs activation. Unstimulated condition and a control oligoribonucleotide with a mutant *let-7b* sequence (i.e., with reduced GU content (28)) served as negative and sequence specificity control, respectively (**Figure 2**).

The exact local concentrations of extracellularly functional miRNA in the brain parenchyma at the site of pathology in humans are not known. Therefore, the miRNA concentrations used in this study were based on our previous work on ssRNA-induced neurodegeneration, where we observed that damaged neurons, and potentially other CNS cells, release miRNAs into the extracellular space and modulate murine microglial function (28, 48). In these previous studies, miRNA concentrations ranged from 1-10 µg/ml. The expression of inflammatory cytokines, including IL-6 and TNF, in iMGLs treated with oligoribonucleotides and TLR agonists was assessed by ELISA and RT-qPCR (**Figures 2A-D**). The concentration of IL-6 protein was significantly increased in the iMGL supernatant in response to extracellular miR-9-5p, miR-132-5p, and miR-340-3p compared to unstimulated control, whereas IL-6 levels in supernatants of iMGLs incubated with miR-30e-3p, miR-501-3p, or *let-7b* did not differ from those detected in the supernatants of unstimulated iMGLs (**Figure 2A**). Also, IL-6 protein concentration was not altered in the supernatant of iMGLs incubated with the mutant oligoribonucleotide compared to the unstimulated condition. Incubation of iMGLs with LPS, R848, or TL8–506 resulted in a significant increase in IL-6 concentrations, whereas loxoribine did not alter IL-6 protein levels compared to the unstimulated control (**Figure 2A**). Among the tested miRNA candidates, only extracellular miR-9-5p increased TNF protein in the supernatant of iMGLs cultures (**Figure 2B**). Similarly, TL8-506, R848, and LPS induced TNF production in iMGLs, whereas loxoribine failed to induce such a response (**Figure 2B**). Analysis by RT-qPCR revealed increased *IL-6*, *TNF* (**Figures 2C, D**), and *IL-1ß* mRNA (data not shown) expression in iMGLs in response to extracellular miR-9-5p and miR-132-5p, but not to miR-30e-3p, miR-501-3p, or *let-7b*, compared to the unstimulated condition. Exposure of iMGLs to miR-340-3p resulted in an increase in *IL-6* and *TNF* (**Figures 2C, D**), but not in *IL-1ß* mRNA (data not shown) expression. In line with our findings on IL-6 protein release, loxoribine and the mutant oligoribonucleotide failed to induce *IL-6* mRNA expression (**Figure 2C**). Altogether, extracellular miR-9-5p, miR-340-3p, and miR-132-5p induced a differentiated cytokine response from iMGLs, whereas *let-7b*, miR-501-3p, and miR-30e-3p failed to induce cytokine release. While both the selective hTLR8 agonist and the dual TLR7/8 agonist induced cytokine release from iMGLs, exposure to the TLR7 agonist loxoribine did not. To further investigate TLR7 function in iMGLs, we employed another TLR7 agonist, namely imiquimod, an imidazoquinoline amine analog to guanosine (86, 89). In contrast to loxoribine, imiquimod was capable of inducing IL-6 release from iMGLs (**Supplementary Figure 1**).

To exclude the impact of cell line-specific effects on miRNA-induced iMGL modulation, we employed iMGLs differentiated from a different iPSC line, namely BJFF.6, in a similar experimental setup as described above (**Supplementary Figure 2**). In line with our findings using the BIHi268-A-10 line, TL8-506, R848, and LPS induced IL-6 release from BJFF.6-derived iMGLs. IL-6 protein concentrations in the supernatants were not increased after incubation with loxoribine or imiquimod (**Supplementary Figure 2A**). Among the tested miRNAs, only extracellular miR-9-5p induced an increase in IL-6 protein secretion compared to the unstimulated condition. Exposure of BJFF.6-derived iMGLs to miR-132-5p and miR-340-3p did not result in a significant IL-6 release, and similarly, IL-6 protein levels did not differ after exposure to miR-501-3p, *let-7b*, and miR-30e-3p from the unstimulated condition (**Supplementary Figure 2A**). Extracellular miR-9-5p, miR-132-5p, and miR-340-3p induced mRNA expression of *IL-6*, *TNF*, and *IL-1ß* in BJFF.6-derived iMGLs. Exposure to miR-501-3p, *let-7b*, and miR-30e-3p did not alter the cytokine mRNA expression levels compared to unstimulated control (**Supplementary Figures 2B-D**). Overall, these data mirror the results obtained from BIHI-268A-10-derived iMGLs, as described above; however, the effects on cytokine expression and production induced by the respective miRNA candidates and TLR agonists were less pronounced. The reduced responses of iMGLs derived from the BJFF.6 line may be due to their lower *TLR8* expression level (see **Figure 1**), suggesting hTLR8 as the receptor responsible for miRNA recognition in iMGLs. Due to the more robust and consistent responses observed in iMGLs differentiated from the BIHi-268-A-10 line, as well as the lower yield of BJFF.6-derived iMGLs, detailed investigations of extracellular miRNA effects on iMGLS were focused on the BIHi-268-A-10 cell line. Both lines were used in initial experiments to exclude donor-specific effects, but practical considerations limited extensive analysis to the more stable BIHi-268-A-10 line.

### Secretion of IL-6 from iMGLs induced by extracellular miRNAs requires TLR8 signaling

**Figure 3:**
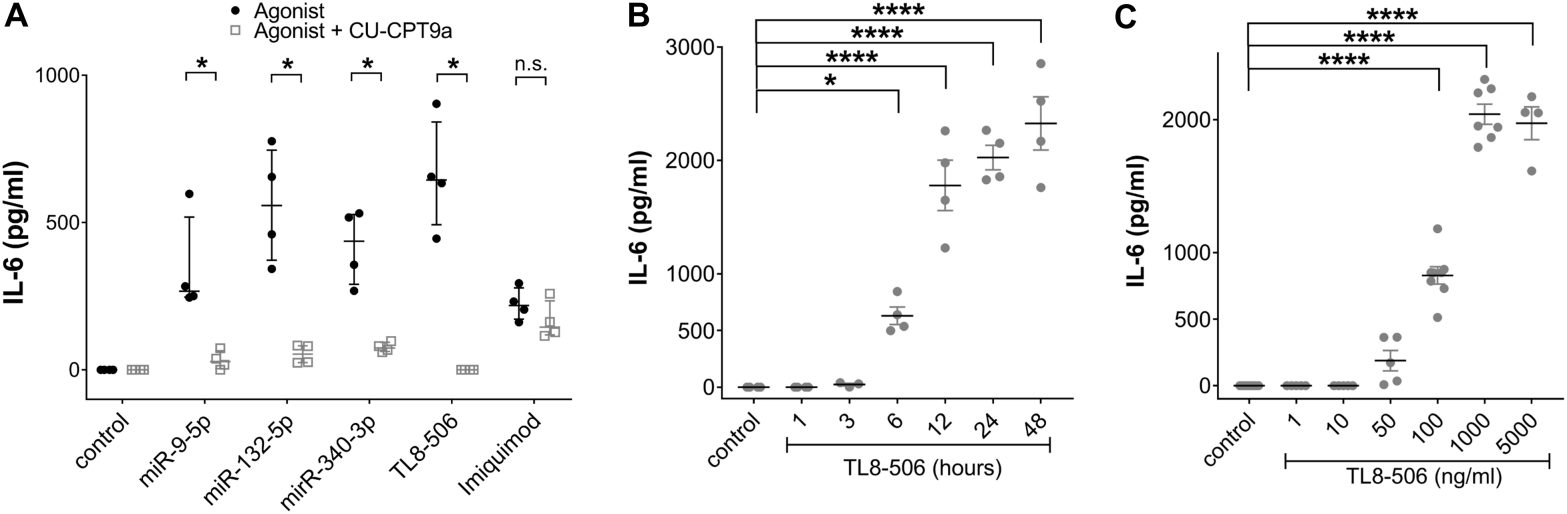
IL-6 secretion from miRNA-treated IMGLs is abolished after inhibition of TLR8 signaling. **(A)** BIHi268-A-10-derived iMGLs were incubated with 10 µg/ml of miRNAs, as indicated. Imiquimod (5 µg/ml) and TL8-506 (100 ng/ml) served as control for TLR7 and TLR8 activation, respectively. Unstimulated cells served as negative control. Assessment of IL-6 protein concentrations after stimulating iMGLs for 24 h, with or without pre-treatment with 1 mM CU-CPT9a for 3 h. Data are shown with the median (bar), interquartile range (whiskers), and single data points (dots). Data were analyzed using the Mann-Whitney-test. *p< 0.05; **p< 0.01; ***p < 0.005; ****p< 0.001, inhibitor pre-treatment was compared to condition without pre-treatment. *n* = 4. **(B)** Assessment of IL-6 protein concentrations after exposing iMGLs to 100 ng/ml TL8–506 for various time periods, as indicated, and **(C)** with different concentrations of TL8-506, as indicated, for 24 h. Unstimulated condition served as negative control. Data are shown as mean (line), SEM (whiskers), and single data points (dots), and were analyzed using a one-way ANOVA followed by Dunnett’s *post hoc* test. *p < 0.05; ****p < 0.001; n.s. = not significant; each condition with inhibitor pre-treatment compared to condition without pre-treatment **(A)**, or compared to unstimulated condition **(B, C)**. n = 4 individual experiments for variation in exposure time and pre-treatment with inhibitor and n = 4-7 for dose response relation.

As described above, miR-501-3p, which activates hTLR7 but not hTLR8 (29, 46), as well as *let-7b*, an established ligand for murine TLR7 (mTLR7) (28, 48), failed to induce IL-6 release from iMGLs. In contrast, miR-9-5p, miR-340-3p, and miR-132-5p, previously identified as hTLR8 ligands (46, 47), triggered cytokine release from iMGLs (see **Figure 2**), suggesting that hTLR8 is the predominant receptor for recognizing extracellular miRNA by human microglia. As we found this receptor to be expressed in iMGLs (see **Figure 1**), we aimed to assess the role of hTLR8 in iMGL cytokine expression induced by extracellular miRNA. To this end, we employed CU-CPT9a, a selective TLR8 inhibitor (78). iMGLs were pre-treated with CU-CPT9a for 3 h and were subsequently exposed to miR-9-5p, miR-132-5p, or miR-340-3p for 24 h, with the inhibitor present in the medium over the whole incubation period. IL-6 concentrations in the iMGL supernatants were subsequently assessed by ELISA (**Figure 3A**). In the presence of CU-CPT9a, the IL-6 release from miR-340-5p-treated cells was significantly reduced, and after miR-9-5p and miR-132-5p treatment, it was abolished, as compared to the control. The TLR8 agonist TL8–506 mimicked the response to the miRNAs by inducing IL-6 production. This IL-6 release was abolished when cells were pre-treated with CU-CPT9a. IL-6 release in response to the TLR7 ligand imiquimod was not affected by pre-incubation with CU-CPT9a, supporting the specificity of CU-CPT9a for hTLR8 (**Figure 3A**). These data support our hypothesis that TLR8, but not TLR7, is mainly responsible for mediating the iMGL response to extracellular miRNA.

To determine the response time for the IL-6 release from iMGLs mediated by hTLR8, we incubated iMGLs with TL8–506 for various time periods, up to 48 h. After 1 and 3 h, no IL-6 release was detected, while after 6 h of TL8–506 exposure, IL-6 concentrations were significantly higher compared to the unstimulated condition. They further increased up to 24 h, reaching a plateau at 48 h (**Figure 3B**).

Next, we assessed whether increased concentrations of TL8–506 result in increased IL-6 release from human microglia. To this end, iMGLs were incubated with TL8–506 at various concentrations for 24 h. Incubation of the cells with 1, 10, and 50 ng/ml did not induce significant IL-6 release. The first significant release of IL-6 was noted when incubating with 100 ng/ml of TL8-506, increased further with 1000 ng/ml of TL8-506, and reached a plateau phase at 5000 ng/ml of TL8-506 (**Figure 3C**). Based on these dose-dependent results, the EC50 for TL8-506-induced IL-6 production was estimated at around 140 ng/ml.

### Distinct extracellular miRNAs affect iMGL motility

**Figure 4:**
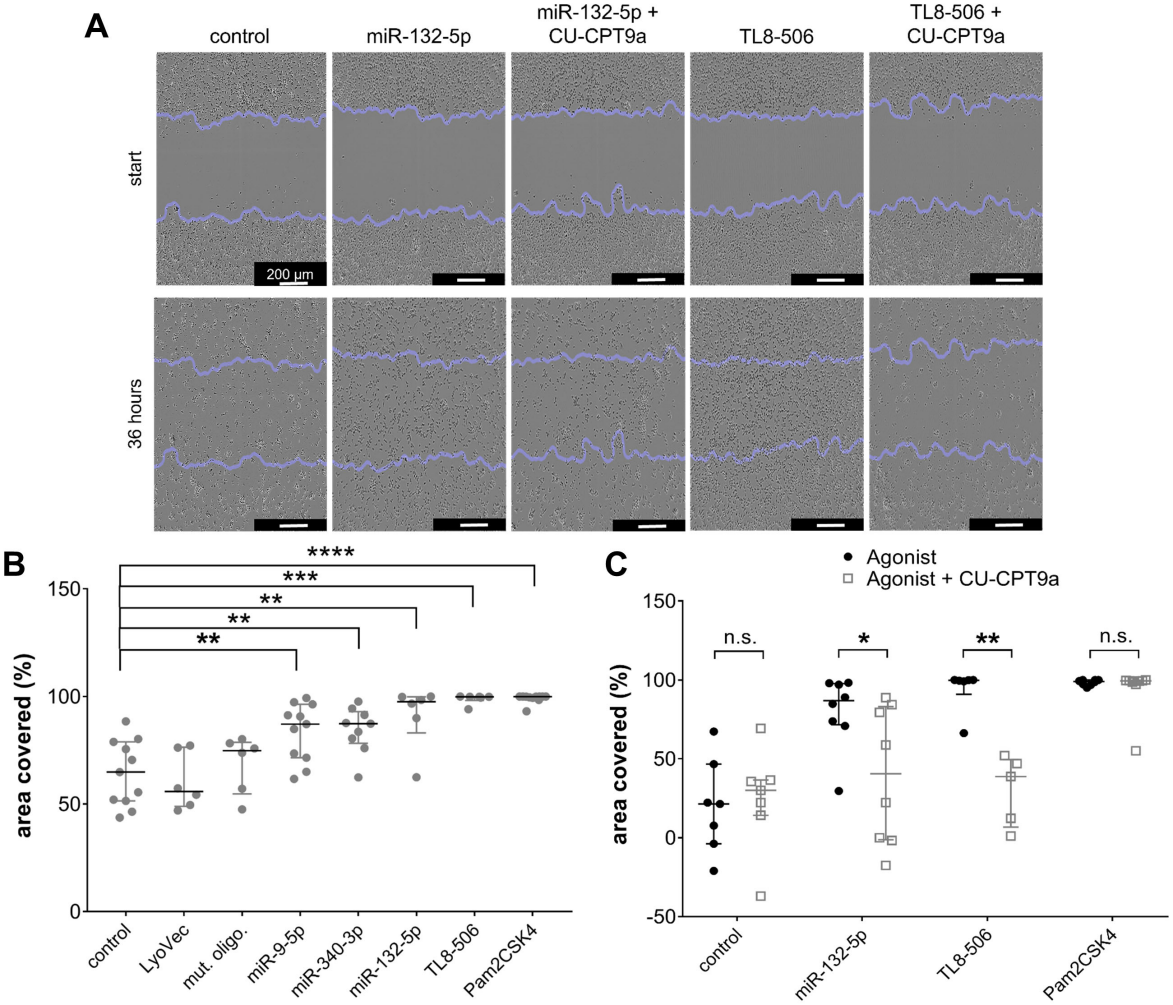
Extracellular miR-9-5p, miR-132-5p, and miR-340-3p induce increase in iMGL motility. **(A-C)** iMGLs were incubated with 10 µg/ml miR-9-5p, miR-132-5p, or miR-340-3p, as indicated, for 36 h. Pam_2_CSK4 (100 ng/ml) and TL8-506 (100 ng/ml) served as positive control for respective TLR activation. Untreated condition, LyoVec, and mutant control oligoribonucleotide (10 µg/ml) served as negative control. Images for motility analysis were acquired with a bright-field microscope. **(A)** Representative images of iMGLs incubated with miR-132-5p (10 µg/ml), Pam_2_CSK4 (100 ng/ml), or TL8-506 (100 ng/ml) with/without CU-CPT9a (1 mM). **(B)** Covered area in percent after incubation of iMGLs with miRNAs and TLR agonists, as indicated, after 36 h. **(C)** Motility rate after incubation of iMGLs with miRNAs and TLR agonists, as indicated, in the presence of CU-CPT9a. Data are shown with median (line), interquartile range (whiskers), and single data points (dots). Data were analyzed using Mann-Whitney-U test, followed by a Benjamini-Hochberg *post hoc* test for multiple testing. *p < 0.05; **p < 0.01; ***p < 0.005; **** *p* < 0.001, n.s. = not significant, compared to unstimulated condition **(B)**, or each condition with inhibitor pre-treatment compared to condition without pre-treatment **(C)**. *n* = 6-11 **(B)** and *n* = 7 **(C)**.

We have demonstrated in previous work that migration of murine microglia is controlled by TLR2 and TLR7 signaling (20, 48). As extracellularly applied miR-9-5p, miR-132-5p, and miR-340-3p were found to trigger cytokine release from iMGLs, predominantly via TLR8, we investigated the impact of these miRNAs on motility. Motility of iMGLs exposed to the miRNAs mentioned above was quantified using a scratch assay (**Figure 4A**), as previously described (75, 79). Representative images of the scratch assay analyzing the iMGLs’ motility in the presence of miRNA and TLR8 agonist with or without TLR8 inhibitor treatment are shown in **Figure 4A**. TL8–506 and Pam_2_CSK4, a TLR2 agonist, increased the motility of murine microglia as previously described (75) and served as positive controls for TLR8- and TLR2-induced activation, respectively, while the mutant oligoribonucleotide served as a control of sequence-specificity. The area recovered by cells due to their migration into the cell-free space was analyzed after 36 h using an ImageJ plugin, as previously described (79). Exposure to miR-9-5p, miR-132-5p, and miR-340-3p increased iMGL motility compared to the unstimulated condition (**Figure 4B**), as well as TL8–506 and Pam_2_CSK4. In contrast, exposure to the sole transfection agent (LyoVec) or the mutant oligoribonucleotide failed to affect iMGL motility compared to the control condition (**Figure 4B**).

To pinpoint the involvement of hTLR8 in modulating iMGLs’ motility induced by the miRNAs, we made use of CU-CPT9a in the experimental setup described above. iMGLs were pre-stimulated with 1 mM of CU-CPT9a for 3 h, and subsequently exposed to miR-132-5p, TL8-506, or Pam_2_CSK4 (**Figure 4C**). After a further 36 h, the area covered with cells was again quantified. We observed reduced iMGL motility after miR132-5p and TL8–506 treatment upon selective hTLR8 inhibition via CU-CPT9a. In contrast, there was no significant change in the motility rate of iMGLs exposed to Pam_2_CSK4 in the presence of CU-CPT9a (**Figure 4C**). These data indicate that the effect of extracellular miR-132-5p and TL8–506 on iMGL motility is mediated via hTLR8.

### Extracellular miR-9-5p, miR-132-5p, and miR-340-3p alter phagocytosis activity of iMGLs

**Figure 5:**
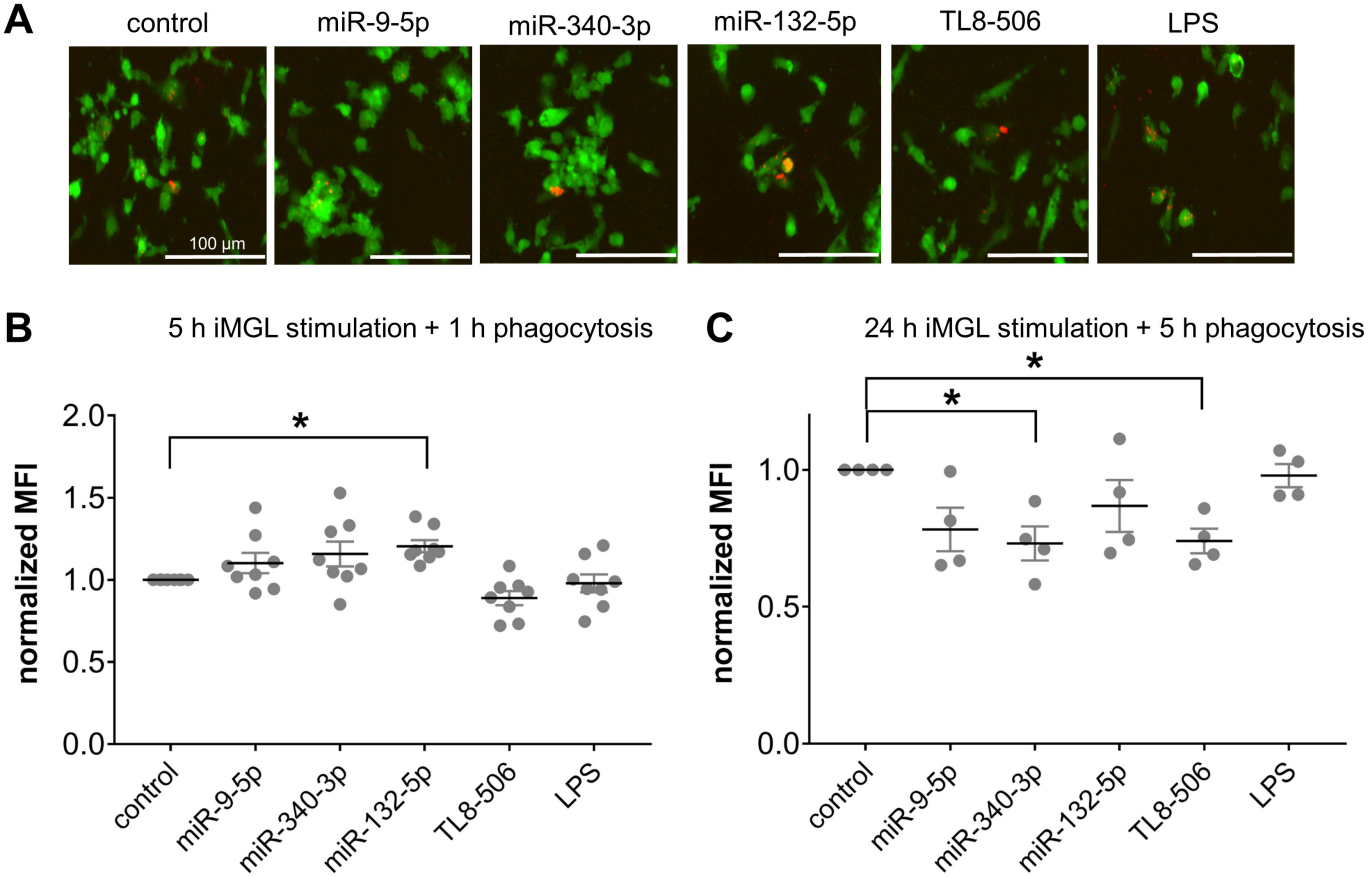
Extracellular miRNA affects phagocytic activity of iMGLs dependent on sequence and time. **(A-C)** iMGLs were incubated with 10 µg/ml miR-9-5p, miR-132-5p, or miR-340-3p, as well as LPS (1 µg/ml) and TL8-506 (100 ng/ml), for 5 h or 24 h, as indicated. Subsequently, phRhodo-labelled *E. coli* particles were added. iMGLs stimulated for 5 h were exposed to *E. coli* particles for 1 h for phagocytosis, while cells stimulated for 24 h were exposed to *E. coli* particles for 5 h. **(A)** Representative images of iMGLs exposed to miRNAs and TLR agonists, as indicated, and incubated with *E. coli* particles for 2 h. **(B)** iMGLs stimulated for 5 h with miRNA und TLR agonists, as indicated, and subsequent 1 h phagocytosis of phRhodo-labelled *E. coli* particles, were analyzed using FACS. Amount of phRhodo-labelled *E. coli* engulfment is shown as mean fluorescent intensity (MFI) normalized to unstimulated control. **(C)** iMGLs were exposed for 24 h to miRNAs and TLR agonists, as indicated, followed by 5 h phagocytosis of *E. coli* particles. Analysis by FACS as described above. Data are shown as mean (line), SEM (whiskers), and single data points (dots), and were analyzed using a one-way ANOVA followed by Dunnett’s test for multiple testing. *p < 0.05. *n* = 8 **(B)** and *n* = 4 **(C)**.

As microglia serve as the primary phagocytes in the CNS upon homeostasis and pathological conditions (90–93), we next investigated whether extracellular miRNAs can affect phagocytosis activity of iMGLs by exposing them to miR-9-5p, miR-132-5p, or miR-340-3p, as well as the TLR4 agonist LPS which has been previously shown to affect phagocytosis activity of murine microglia (94, 95), and TL8-506. Based on previous work (75, 77), we exposed iMGLs for 5 h or 24 h to miRNAs, followed by an incubation with pHrodo™ Red *E. coli* BioParticles™ for 1 h or 5 h, respectively (**Figure 5**). iMGLs containing *E. coli* BioParticles™ (**Figure 5A**) were quantified by FACS (gating strategy shown in **Supplementary Figure 3**), and the extent of their phagocytic activity was represented by mean fluorescence intensity normalized to the control condition (normalized MFI; **Figures 5B, C**). We observed a slight, but statistically significant, increase in the phagocytic activity of iMGLs exposed to miR-132-5p for 5 h and incubated with *E. coli* particles for a further 1 h. No such changes in iMGL phagocytosis were detected after incubation with miR-9-5p or miR-340-3p (**Figure 5B**). In contrast to these results, exposure to miR-340-5p and TL8–506 for 24 h, followed by 5 h of incubation with pHrodo™-labelled *E. coli* BioParticles™, led to a significant reduction of iMGL phagocytosis (**Figure 5C**). Taken together, extracellular miRNAs modulate iMGL phagocytosis dependent on their sequence and exposure time.

### Extracellular miR-132-5p and miR-9-5p reduce neurite length in co-cultures of iPSC-derived human cortical neurons and iMGLs

**Figure 6:**
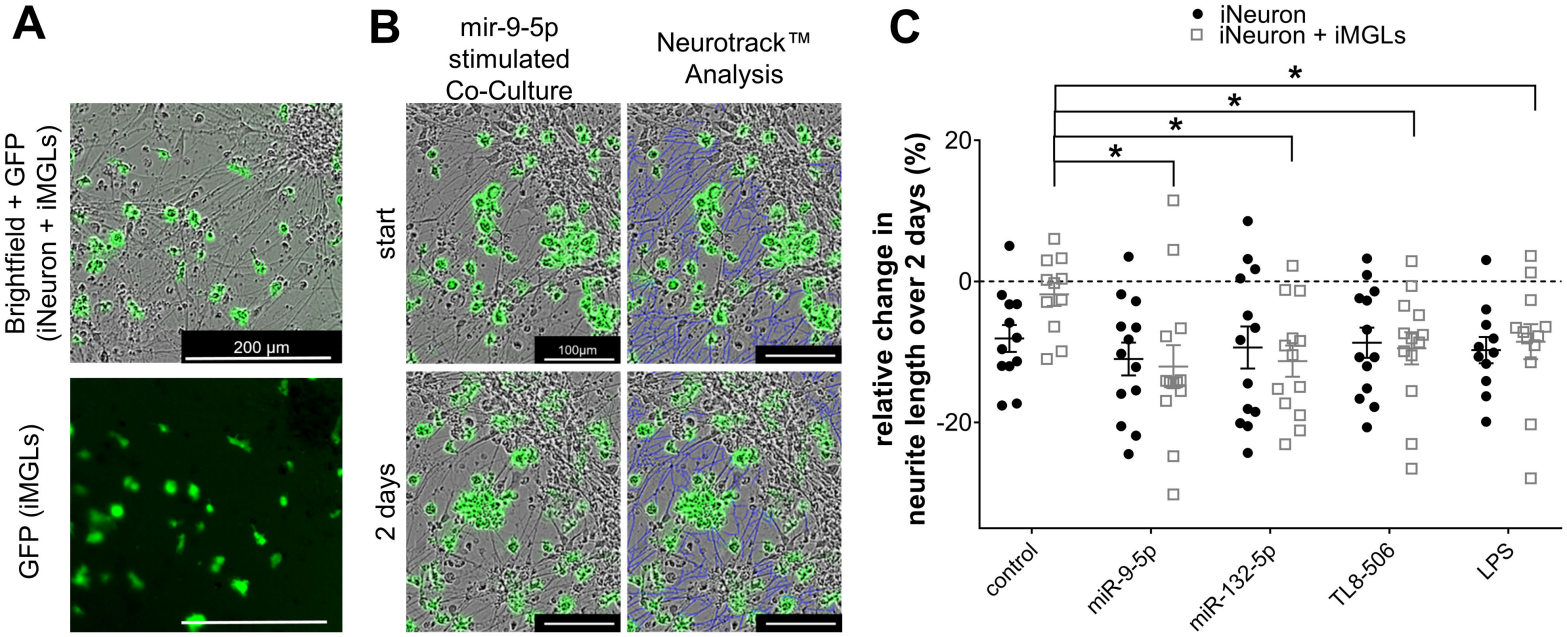
Extracellular miR-9-5p and miR-132-5p induce iNeuron neurite length reduction. **(A)** Representative images of co-cultures containing iNeurons and GFP-positive iMGLs (top, bright field). The fluorescence image at the bottom depicts the same field of view illustrating the distribution of the GFP-positive iMGLs. **(B)** Representative images of co-cultures containing iNeurons and iMGLs incubated with miR-9-5p at the beginning of exposure and after 2 d (left), analyzed by the Neurotrack*™* software (neurites indicated in blue color, right). **(C)** Assessment of relative change in neurite length of iNeurons co-cultured with iMGLs and iNeuron monocultures, as indicated, after 2 d exposure to 10 µg/ml miR-9-5p, or miR-132-5p. LPS (1 µg/ml) and TL8-506 (100 ng/ml) were tested in parallel. Unstimulated condition served as negative control. Data are shown with mean (line), SEM. (whiskers), and single data points (dots), and were analyzed using a one-way ANOVA followed by Tukey’s *post hoc* test to test for significant difference between stimulated and unstimulated condition, as indicated. *p < 0.05; *n* = 11-13.

In mouse models, various TLRs, including TLR2, TLR4, and TLR7, affect neuronal structure and viability. While some of these effects operate in a cell-autonomous fashion, some are mediated by microglia (28, 48, 96, 97). To determine whether extracellular miRNAs affect neurons in a human *in vitro* system, iMGLs were co-cultured with iPSC-derived human cortical neurons (iNeurons), which were differentiated based on an established protocol (80). In short, the iPSC line BIHi005-A-24 overexpressing doxycycline-inducible neurogenin-2 was differentiated into functional cortical neurons over the course of two weeks. Subsequently, iMGLs were added to the iNeuron culture in a ratio of 1:3 (iMGLs:iNeurons) (**Figure 6A**). After a further 24 h, co-cultures of iNeurons and iMGLs were exposed for 2 d to miR-132-5p or miR-9-5p, which were found to potently modulate iMGL functions in our study (see **Figures 2**, **4**, **5**). During the miRNA exposure period, images of iNeurons and co-cultures of iNeurons and iMGLs were taken every 24 h and analyzed employing the Incucyte’s analysis module Neurotrack (98). In this analysis, the length of a given iNeuron neurite at each individual time point was assessed as indicated by the representative images of a co-culture exposed to miR-9-5p (**Figure 6B**). To determine the effect of the extracellular miRNAs and the TLR4 and TLR8 agonists LPS and TL8-506, respectively, over time and to accommodate the high variability between the individual cell cultures, the relative change in neurite length was subsequently analyzed using the following equation: $\frac{{\text{l}}_{\text{x }}-l_{0}}{l_{0}}*100$, in which l_x_ stands for neurite length after 2 d and l_0_ equals neurite length at the start of the experiment. Incubation of co-cultures containing iNeurons and iMGLs with miR-132-5p and miR-9-5p led to a significant reduction of neurite length over the course of 2 d by 11.3% and 12%, respectively, compared to the unstimulated condition. Incubation with TL8–506 and LPS resulted in a reduction of the neurite length by 9.5% and 8.5%, respectively (**Figure 6C**). The neurite length in pure iNeuron cultures lacking iMGLs exposed to miR-9-5p, miR-132-5p, LPS or TL8–506 did not differ from the unstimulated condition, indicating that iMGLs are required for the observed changes in neurite length of iNeurons (**Figure 6C**).

## Discussion

Microglia, as the resident immune cells in the brain, detect pathogens and signals of a disturbed homeostasis. These signals alter microglial function and are sensed in part by TLRs (4, 7, 9, 14, 99). Consistent with a central role for these receptors in brain homeostasis, miRNAs have been recently discovered as ligands for TLRs (28, 29, 46, 47, 100). Selected miRNAs directly activate TLR7 expressed in murine microglia (48); however, the role of miRNAs as ligands for TLR7/8 in the human brain has not been analyzed so far. Here, we investigated whether human microglial function is affected by extracellular miRNA. To this end, we generated human microglia-like cells from induced pluripotent stem cells and confirmed mRNA expression of both *TLR7* and *TLR8*, the two miRNA-sensing receptors identified so far besides TLR1 (30, 120). We then assessed whether selected miRNAs, which i) based on their sequence and structure have been previously predicted as potent hTLR7 and/or hTLR8 activators by the algorithm *Braindead*, ii) have been validated as ligands for these RNA sensors, and iii) whose expression is specifically dysregulated in AD and glioma, can act as extracellular ligands, thereby altering iMGL function. Besides *let-7b*, which is an established ligand for mTLR7 and contributes to neurodegenerative processes in the murine CNS (28, 48), the miRNA candidates of our current study included miR-9-5p, miR-132-5p, miR-340-3p, miR-30e-3p, and miR-501-3p. iMGLs responded to a subset of these miRNAs with cytokine release, enhanced migration, and an altered phagocytic activity. The findings reported herein raise several important questions and aspects regarding human microglial function and interspecies differences.

Our prime goal was to confirm the previous finding from murine cells in human iMGLs, namely that extracellular, disease-associated miRNAs act as signaling molecules through TLR7 and TLR8. We identified miR-9-5p, miR-132-5p and miR-340-3p as modulators of iMGLs differentiated from two different iPSC lines, as indicated by their ability to induce elevation of cytokine protein and/or mRNA levels. In contrast, miR-30e-3p, miR-501-3p and *let-7b* did not induce cytokine secretion. All three miRNAs, miR-9-5p, miR-340-3p, and miR-132-5p, have previously been described as ligands for both hTLR8 and hTLR7 (29, 46, 47). In contrast, those miRNAs that did not affect iMGL functions in our study have been shown to lack the ability of hTLR8 activation (miR-501-3p) or are known activators of TLR7, but not TLR8 (*let-7b*) (28, 46–48). Unexpectedly, miR-30e-3p did not modulate iMGL function, although it was capable of hTLR8 activation in HEK TLR reporter cells (46). Similar to the miRNAs acting extracellularly on iMGLs, the selective TLR8 agonist TL8-506, but not the TLR7 agonist loxoribine, induced cytokine expression, increased motility, and modulated phagocytosis of iMGLs. Remarkably, imiquimod, another established TLR7 agonist that also interferes with adenosine receptor signaling pathways (101), induced IL-6 secretion from BIHi268-A-10-derived iMGLs. This response may be due to a higher binding affinity to the receptor since the chemical TLR7 or TLR7/8 agonists such as imiquimod or resiquimod 848, respectively, have stronger binding capabilities than guanosine analogs like loxoribine (102). Given that in our study, i) inhibition of TLR8 resulted in both diminishing cytokine secretion and motility induced by extracellular miRNA, and ii) TL8-506, but not loxoribine, activated iMGLs, we conclude that extracellular miRNAs modulate human microglial function through TLR8 but not TLR7, which represents the major miRNA sensor in the mouse brain (28).

This observation regarding receptor specificity raises questions about the species-specific differences of TLR 7/8 signaling. Notably, the TLR family exhibits remarkable species differences, starting with the number of functional TLRs: 13 in mice and 10 in humans (16). Evolutionary studies suggest that nucleic-acid-sensing TLRs, particularly TLR8, have undergone strong selective pressures (103). Given the implication of TLR7 in autoimmune diseases (30, 104, 105), it is interesting to speculate that an attenuation of TLR7 signaling was necessary, leaving TLR8 to emerge as the primary miRNA-sensing TLR in humans. Indeed, miRNA-induced NF-κB activation is stronger in murine TLR7 than in human TLR7 in HEK TLR reporter cells (47). At this point, due to a lack of ligands/agonists for mTLR8, the functional relevance of both mTLR8 and its interaction with mTLR7 is unclear (18), and future studies are needed to illuminate the species-specific differences in TLR7 and TLR8 signaling.

We also observed differences between iMGLs derived from the iPSC line BIHi-268-A-10 (female) and BJFF.6 (male). Both responded to the same miRNAs, but cytokine levels and *TLR8* expression levels were higher in BIHi-268-A-10-derived cells. However, *TLR7* expression levels were not significantly different. Given that *TLR7* and *TLR8* are X-linked genes that escape X-inactivation in immune cells (106, 107), sex-related dosage effects are possible. Still, one would have expected a similar increase in *TLR7* and *TLR8* expression levels in the BIHi-268-A-10 line. The observed difference in these expression levels might result from donor variability, differences in gene regulation, or varying escape from X-inactivation. To establish that the observed differences are due to sex difference, future studies analyzing large cohorts of male and female lines to exclude the influence of donor variability or differentiation protocols are required. Such investigations may include transcriptomic profiling and a broader set of iPSC lines from both sexes (104, 108–110).

Another major goal of this study was to characterize in detail the changes in human microglial function induced by extracellular, disease-associated miRNA. Different miRNAs elicited distinct cytokine responses when applied extracellularly. IL-6 release was observed in response to miR-9-5p, miR-132-5p, and miR-340-3p; no such response was observed after exposure to *let-7b*, miR-30e-3p, or miR-501-3p. Among the tested miRNAs only miR-9-5p induced TNF release from iMGLs, which is in contrast to our previous studies on mouse microglia releasing TNF in response to all of the miRNAs mentioned above (29, 47, 48), however it aligns with previous observations that specific miRNAs can induce individual cytokine patterns in murine microglia (29, 48). These differences in human and mouse studies may be due to cell type-specific responses, particularly as other groups have previously demonstrated that stimulated iMGLs predominantly express IL-6 (82).

In our current study, those miRNAs that induced cytokine expression, namely miR-9-5p, miR-340-3p, and miR-132-5p, also modulated iMGL motility. These miRNA-induced effects were sequence-specific, as the other tested miRNAs failed to affect iMGL motility. However, in contrast to iMGLs, murine microglia are attracted by both miRNA and mutant oligoribonucleotides with scrambled sequence (48). These different findings in mouse and human studies imply that migration of mouse microglia is modulated by small ssRNA molecules in general but is not dependent on sequence, while human microglial motility seems to be regulated by distinct miRNAs in a sequence-specific fashion, indicating species-specific differences with respect to the miRNAs’ function as signaling molecules for microglia. Moreover, select miRNAs induced opposite effects on iMGLs. While extracellular miR-132-5p increased the iMGLs’ phagocytic activity, miR-340-3p diminished phagocytosis, and in contrast, extracellular miR-9-5p did not affect iMGL phagocytosis. Considering that the mutant oligoribonucleotide had no impact on any of the iMGL functions investigated in this study, these data support our postulate that miRNAs acting as signaling molecules for human microglia operate strictly in a sequence-specific fashion.

Similar to *let-7b*, which possesses a sequence comprising the established mTLR7 recognition motif GUUGUGU (28, 48), miR-9-5p, miR-132-5p, and miR-340-3p, which modify iMGLs via hTLR8, contain GU-rich motifs known to activate both hTLR7 and hTLR8 with minimal exchanges of nucleotides (29, 111, 112). Our findings on miRNA-induced iMGL modulation are in accordance with previous studies, in which miR-340-3p and miR-132-5p induce cytokine release from human macrophages (47), and the sequence UUGU was identified as the minimum motif required for TLR8- and TLR7-mediated cytokine responses from human peripheral blood mononuclear cells (111). This selectivity is important, as it supports a model in which different miRNAs have unique roles in modulating microglial function in the human brain. However, while we did not observe iMGL responses to extracellular miR-501-3p, which does not contain any of the established sequence motifs required for hTLR7/8 activation and lacks the ability to activate hTLR8 in HEK TLR reporter cells (46), miR-30e-3p, comprising GU-rich recognition motifs, was not capable of altering iMGL functions either. Thus, further studies are necessary to elaborate the miRNAs’ detailed sequence and structure features required for hTLR8 binding and activation, and thereby modulating human microglial function.

In the murine CNS, injured neurons and possibly other CNS cell populations release miRNAs into the extracellular space, thereby triggering neurodegenerative processes (29). In line with this, miR-340-3p and miR-132-5p, which stimulated iMGLs in our current study, have been previously shown to be released from murine apoptotic neurons, to be extracellularly stable, and to induce further injury of murine neurons when extracellularly applied (29, 47). Whether cellular-specific miRNAs passively leak into the extracellular space of the CNS or are actively secreted in their native state or enclosed in vesicles remains unresolved. For example, lung tumor cells release miR-21 and miR-29a within exosomes, and these miRNAs can be phagocytosed by macrophages and activate both mTLR7 and hTLR8 (42). Whether such miRNA release and uptake mechanisms play a role in the modulation of microglial function by extracellular miRNA in a pathophysiological context, particularly in the human brain, requires further investigation.

Our results extend the physiological role of miRNAs beyond their established role in the regulation of gene expression to ligand-mediated activation of receptors in human microglia. As such, specific miRNAs act as ligands for TLRs in the human brain, where they may modulate and contribute to CNS pathologies, such as neurodegeneration, tumor growth, and immune responses, among others. In neurodegenerative diseases, the contribution of neuroinflammation driven by microglial alteration is well established (4, 7, 9, 113). Our results imply that extracellular miRNAs such as miR-9-5p and miR-132-5p, both dysregulated in AD and glioma, are not only able to modulate microglial function, possibly contributing to maladaptive microglial states, but also can affect human neurons. Although the exact mechanism underlying the neurite length alteration in our human co-culture system remains unidentified, it is tempting to speculate that the neurite length reduction by miR-132-5p and miR-9-5p was mediated by yet unidentified factors released from iMGLs, such as cytokines, chemokines, and/or reactive oxygen species (ROS). Extracellularly applied miRNAs may have specifically reprogrammed microglial cytokine release, possibly also their migration and phagocytosis activity, thereby affecting neuronal structure.

The convergence of miRNA dysregulation in two distinct diseases, AD and glioma, suggests that altered miRNA-microglia signaling represents a fundamental mechanism rather than a disease-specific pathway. This is further supported by evidence that aging skews miRNA expression toward a pro-inflammatory profile (114), potentially amplifying their impact in age-related disorders. Furthermore, miRNAs and TLR signaling are emerging as promising diagnostic and therapeutic targets in neurological disorders. For example, antagomirs, synthetic inhibitors of miRNAs, are being investigated for therapeutic gene regulation. Understanding the diverse functions of miRNAs, including their role as signaling molecules, is critical for developing safe and effective interventions and minimizing off-target effects (43, 49, 54–56, 115).

This study has various limitations that need to be acknowledged. First, the effects of miRNAs on human microglia and neurons observed *in vitro* can be over- or underestimated in comparison to the *in vivo* situation since the precise on-site concentrations of extracellular miRNA in the brain in a pathophysiological context are not known. Second, even though human iMGLs share key characteristics with human microglia, such as transcriptome, functional properties, and maturation marker expression, they more closely resemble fetal human microglia (75, 82, 116). Therefore, age-related changes in microglial function might not be fully represented in our experimental model. Also, employing two iPSC lines in this study makes future studies with a more extensive and diverse selection of iPSC lines necessary. Lastly, our *in vitro* study analyzed iMGL monocultures and co-cultures of iMGLs and iNeurons. It should be noted that our co-culture system contained BIHi005-A-24–derived neurons (male donor) and BIHi268-A-10–derived microglia (female donor). While potential confounding effects arising from the combination of cell lines of different sexes and donor origin cannot be entirely excluded, alternative pairings were not feasible due to limited differentiation capacity of the available iPSC lines in our study. Importantly, the control unstimulated co-cultures remained viable and stable throughout the experiments, and the setup was suitable to address our main objective, namely, to assess whether miRNA-induced human microglia modulation could exert detrimental effects on neurons. Further studies on more complex models comprising further CNS cells and structures, such as astrocytes, adaptive immune cells, and the vascular compartment, are needed to fully understand the complex role of miRNAs acting as signaling molecules in the CNS. However, our study offers novel insight into the role of miRNAs as signaling molecules for human iMGLs and highlights species-specific differences in TLR7/8 signaling that have been intensely discussed in the past (27, 117–119). Unlike patient-derived microglia, which can be altered by disease or treatment history, iPSC-derived microglia used here offer a more controlled and physiologically human model system (9, 10, 116).

Based on our current and previously published data, we propose that certain miRNAs act as endogenous ligands for hTLR8. The selective modulation of human microglial function observed here suggests that selected miRNAs, and perhaps other miRNA-related RNA species, elicit a specific reprogramming of the cells that promotes specific physiological processes important for brain homeostasis and certain human disease states. To conclude, we demonstrate that human CNS disease-related miRNAs function as signaling molecules for human microglia in a sequence-specific fashion via TLR8, thereby modulating diverse microglial functions and affecting neuronal structure. A greater understanding of the extracellular miRNAs’ role as fine-tuning modulators of human CNS cell function, deciphering the involved signaling pathways, and determining the posttranscriptional changes in the different CNS cell types, including glial cells and neurons, may reveal potential roles for these small RNAs as novel therapeutic targets in various human CNS diseases.

## Data Availability

The raw data supporting the conclusions of this article will be made available by the authors, without undue reservation.
