# Supplementary material for: Extracellular microRNAs modulate human microglial function through TLR8
Source: Front Immunol. 2025 Nov 14;16:1645062. doi: 10.3389/fimmu.2025.1645062 (PMC12660282; doi:10.3389/fimmu.2025.1645062)
Supplement: Supplementary file 1 [file Table1.docx]

Supplementary Material

# Supplementary Figures and Tables

## Supplementary Figures


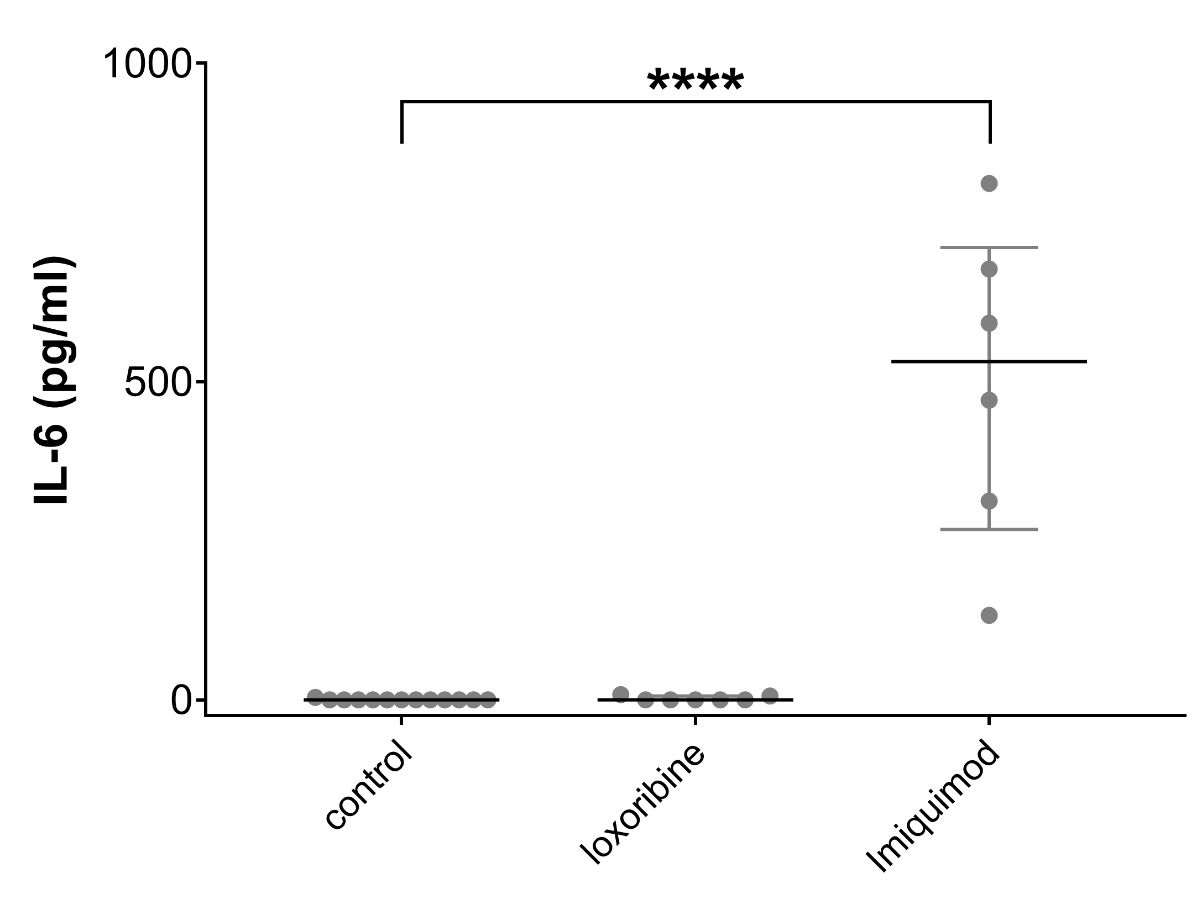


**Supplementary Figure 1. Effect of TLR7 agonists on IL-6 release from iMGLs differentiated from the BIHi268-A-10 line.** iMGLs generated from the BIHi268-A-10 line were stimulated with either loxoribine (1 mM) or imiquimod (5 µg/ml) for 24 h, as indicated. Unstimulated condition served as negative control. Data are shown as a median (line) with an interquartile range (whiskers) and single data points (dots). Kruskal-Wallis test followed by Dunn’s post-hoc multiple comparisons test compared to unstimulated condition. *n =* 6-13.


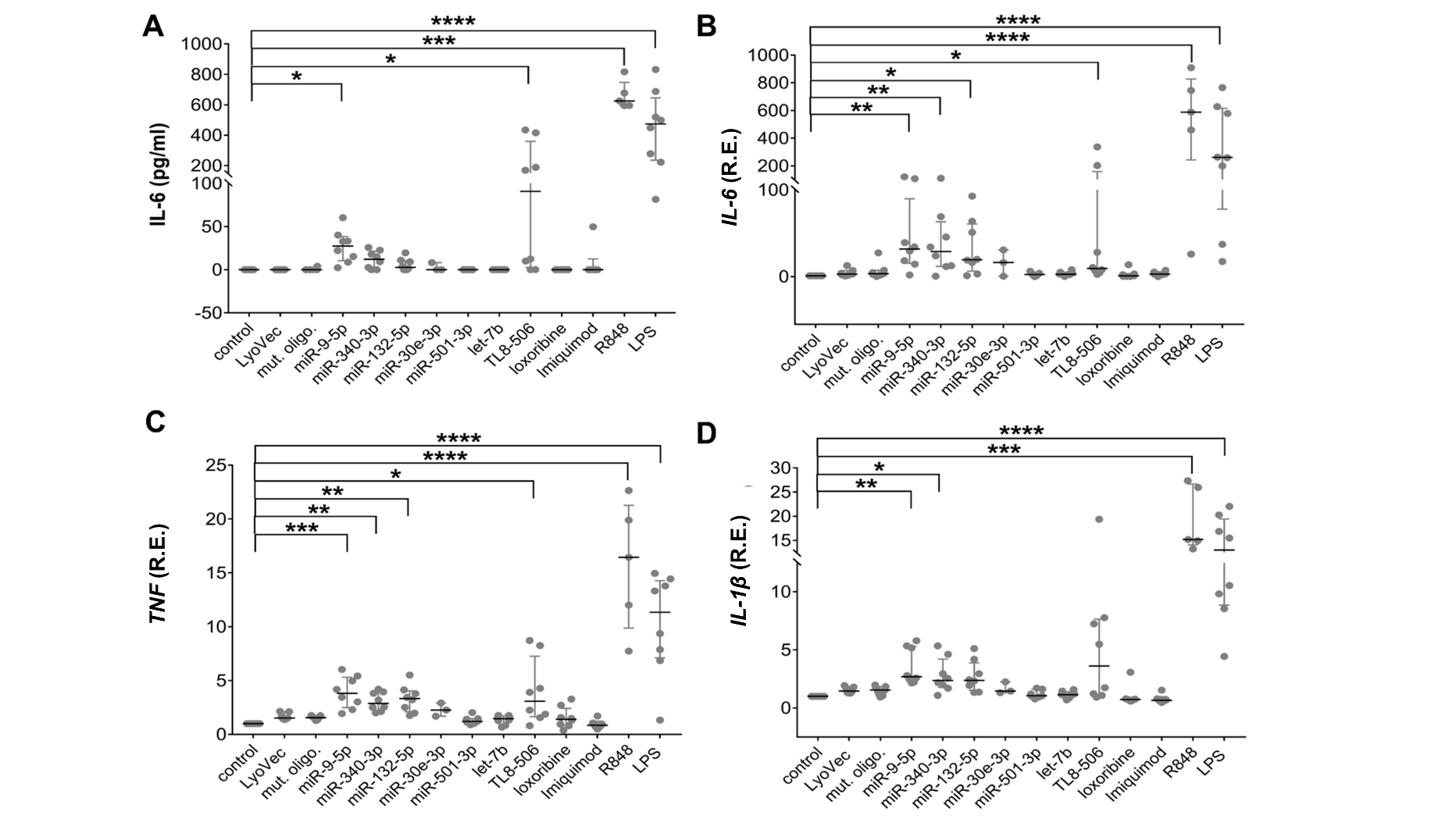


**Supplementary Figure 2. Effects of extracellular miRNAs on cytokine production of BJFF.6-derived iMGLs. (A-D)** BJFF.6-derived iMGLs were exposed to miRNAs and TLR agonists, as indicated, for 24 h. **(A) Assessment of IL-6** protein concentrations in the iMGL supernatant by ELISA. *IL-6* (**B**), *TNF* (**C**), and *IL-1β* (**D**) mRNA expression by qPCR, after miRNA and TLR agonist treatment, as indicated. LPS (1 µg/ml), loxoribine (1 mM), imiquimod (5 µg/ml), R848 (10 µg/ml), and TL8-506 (100 ng/ml) served as positive control for TLR7, TLR7/8, and TLR8 activation, respectively. Unstimulated condition, LyoVec, and mutant oligoribonucleotide (10 µg/ml) served as negative control. Data are shown as a median (line) with an interquartile range (whiskers) and single data points (dots). Kruskal-Wallis test followed by Dunn’s post-hoc multiple comparisons test compared to unstimulated condition and Student’s *t*-test with Benjamini-Hochberg post-hoc correction. **p* < 0.05; ***p* < 0.01; ****p* < 0.005; *****p* < 0.001, compared to unstimulated condition. *n* = 3-8.


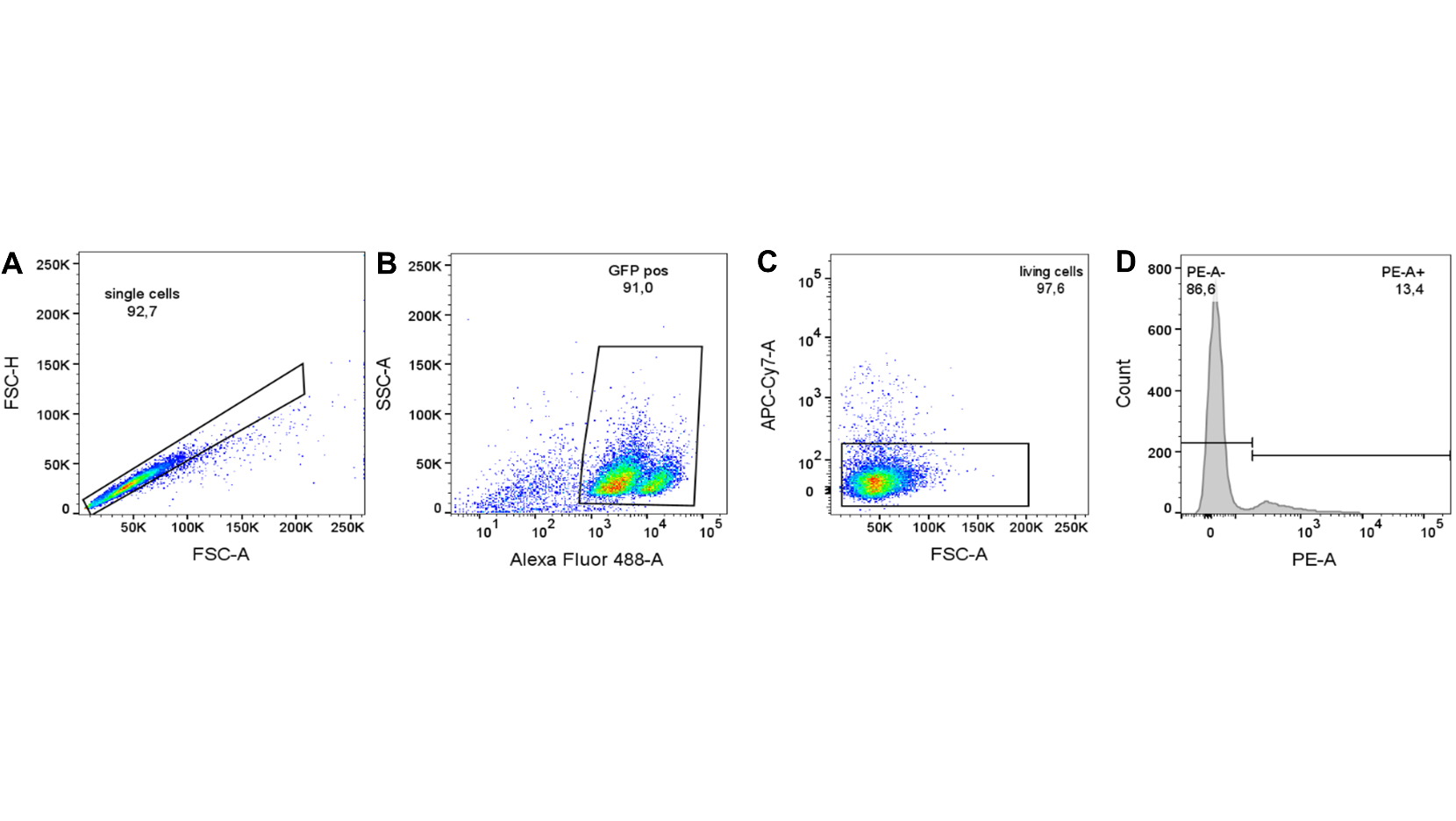
**Supplementary Figure 3. Gating strategy for FACS-based phagocytosis analysis.** In the first step, only iMGLs as single cells were included in the analysis (**A**). Next, GFP-positive cells were included, as iMGLs derived from this cell line express GFP intrinsically (**B**). Finally, only live cells negative for zombie staining (APC-negative) were included in the analysis (**C**). Analysis was performed on PE-positive cells (i.e. cells with phagocytosed phRhodo *E. coli* particles, PE-A).

## Supplementary Tables

**Supplementary table 1. Overview primer sequences.**

| Target | Forward primer 5’-3’ | Reverse primer 5’-3’ |
| --- | --- | --- |
| *TNF* | ATGAGCACTGAAAGCATGATCC | GAGGGCTGATTAGAGAGAGGTC |
| *IL-6* | AACCTGAACCTTCCAAAGATGG | TCTGGCTTGTTCCTCACTACT |
| *IL-1β* | CAGCTACGAATCTCCGACCAC | GGCAGGGAACCAGCATCTTC |
| *TLR7* | GGACTCACTTATTTAAAATCCCTTT | GCAATTTCCACTTAGGTCAAGA |
| *TLR8* | AACTGCCAAGCTCCCTACG | CAAGGCACGCATGGAAATGG |
| *TBP* | AGCGCAAGGGTTTCTGGTTT | CTGAATAGGCTGTGGGGTCA |

**Supplementary table 2. Overview reagents.**

| material | company | identifier |
| --- | --- | --- |
| ⍺-Thioglycerol | Merck | M1753-100ML |
| 96 Well glass bottom plates | Cellvis | P96-1.5P |
| B-27 Supplement (50X), serum free | Life Technologies | 17504-044 |
| Bambanker | GC Lymphotec | 302-14681 |
| bFGF | PeproTech | 100–18B |
| Recombinant human CD 200 | Bon Opus Biosciences | BP004-50ug |
| Cell culture plate, 96 well, surface: Standard, flat base | Sarsted | 83.3924 |
| CU-CPT9a | Invivogen | inh-cc9a |
| CX3CL1, 50µg | Peprotech | 300-31-50UG |
| Cytarabine | Sigma Aldrich | C1768-100MG |
| DMEM/F-12, HEPES | Life Technologies | 11330032 |
| DMEM/F-12, HEPES, no phenol red | Life Technologies | 11039-021 |
| Doxycyclin -hydrochlorid | Sigma Aldrich | D3447-500MG |
| Falcon® 6-well Clear Flat Bottom Plates | Corning | 353046 |
| Geltrex™ | Life Technologies | A14133-02 |
| GlutaMAX Supplement-100 mL | Life Technologies | 35050-038 |
| HEPES | ThermoFisher Scientific | 15630080 |
| Human Insulin | PromoCell | C-52310 |
| insulin | CS Bio, or Sigma, | C9212-1G or 91077C-1G |
| Human NT-3 | R&D Systems | 267-N3-025 |
| human transferrin) | Sigma | T3705-1G |
| IL-34 100µg | Peprotech | 200-34-100UG |
| Imiquimod (R837) | Invivogen | tlrl-imqs-1 |
| Incucyte® Imagelock 96-well plates | Sartorius | BA-04856 |
| Insulin-Transferrin-Selenite | Life Technologies | 41400-045 |
| Mouse Laminin | Sigma Aldrich | L2020-1MG |
| L-ascorbic acid 2-phosphate | Sigma-Aldrich | #A8960 |
| Lipopolysaccharide from E.coli | Merck | L43191 |
| Loxoribine | Invivogen | tlrl-lox |
| LyoVec™ complexer | Invivogen | https://www.invivogen.com/ |
| M-CSF | Peprotech | 300-25-50UG |
| MEM Non-Essential Amino Acids Solution (100X)-100 mL | Life Technologies | 11140-035 |
| TLR2 Agonist Pam2CSK4 | R&D Systems | 4637/1 |
| N-2 Supplement (100X) | Life Technologies | 17502-048 |
| Neurobasal™ Medium | ThermoFisher Scientific | 21103049 |
| PBS | Life Technologies | 14190-169 |
| pHrodo™ BioParticles™ Conjugates for Phagocytosis and Phagocytosis Kit, for Flow Cytometry | ThermoFisher Scientific | P35361 |
| Recombinant Human BDNF | Peprotech | 450-02-100UG |
| Resiquimod | Invivogen | tlrl-r848-1 |
| StemMACS iPS-Brew XF | Miltenyi Biotec | 130-107-086 |
| StemPro® Accutase® | Life Technologies | A11105-01 |
| sodium bicarbonate 7.5% solution | Fisher Scientific | #25080–094 |
| sodium carbonate 0.1M | Carl ROTH | A135.1 |
| sodium selenite | Sigma | #S5261-10G |
| SYBR Green Master Mix | Life Technologies | 4472918 |
| TGFβ1 | Peprotech | 100-21C-50UG |
| TL8-506 | InvivoGen | Tlrl-Tl8506 |
| TLR2 Agonist Pam2CSK4 | R&D Systems | 4637/1 |
| Thiazovivin | StemCell Technologies | 72252 |
| TrypLE™ Select Enzyme (1X), no phenol red | Thermo Fisher | 12563011 |
| Zombie NIR™ fixable dye | BioLegend | 423105 |
| GenePrint® 10 System | Promega | B9510 |
| human IL-6 Uncoated ELISA Kit | ThermoFisher Scientific | 88-7066-88 |
| Human TNF alpha Uncoated ELISA Kit | ThermoFisher Scientific | 88-7346-77 |
| PrimeScript RT reagent kit | Takara Bio | RR037B |
| ReliaPrep™ RNA Tissue Kit | Promega | Z6112 |
| STEMdiffTM Hematopoietic Kit | Stemcell Technologies | 5310 |
| Venor®GeM qOneStep, | Minerva Biolabs | 11-91025 |
| hsa-miR-9-5p  5’-UCUUUGGUUAUCUAGCUGUAUGA-3’ | Integrated DNA Technology | http://www.idtdna.com/pages |
| hsa-miR-340-3p  5’-UCCGUCUCAGUUACUUUAUAGC—3’ | Integrated DNA Technology | http://www.idtdna.com/pages |
| hsa-miR-132-5p  5’-ACCGUGGCUUUCGAUUGUUACU-3’ | Integrated DNA Technology | http://www.idtdna.com/pages |
| hsa-miR-30e-3p  5’-CUUUCAGUCGGAUGUUUACAGC-3’ | Integrated DNA Technology | http://www.idtdna.com/pages |
| hsa-miR-501-3p  5’-AAUGCACCCGGGCAAGGAUUCU-3’ | Integrated DNA Technology | http://www.idtdna.com/pages |
| hsa-let-7b  5’-UGAGGUAGUAGGUUGUGUGGUU-3’ | Integrated DNA Technology | http://www.idtdna.com/pages |
| mutated oligonucleotide  5’-UGAGGUAGAAGGAUAUAAGGA-3’ | Integrated DNA Technology | http://www.idtdna.com/pages |
| Fiji ImageJ software | NIH | https://imagej.nih.gov/ij/download.html; RRID: SCR_003070 |
| \| GraphPad Prism 9 \|  \| \| --- \| --- \| | GraphPad | RRID:SCR_002798 |
| FlowJo v10 software | LLC, BD Life Sciences | http://docs.flowjo.com/d2/ |
| Microsoft Excel | Microsoft | https://www.microsoft.com/en-us/; RRID:SCR_016137 |
| Incucyte NeuroTrack Analysis Software | Sartorius | RRID:SCR_025411 |
| Essen Incucyte | Sartorius | RRID:SCR_019874 |
| Leica DMi8 inverted microscope | Leica | RRID:SCR_026672 |
| \| FACS Aria II flow cytometer \| \| --- \| | BD Bioscience | \| N/A \|  \| \| --- \| --- \| |
| Varioskan Flash device | Thermo Fisher Scientific | N/A |
| NanoDrop 8000 spectrometer | Thermo Fisher Scientific | ND8000LAPTOP |
| 7500 Fast Real-Time PCR System | Applied Biosystems | N/A |
